# Effect of the genetic and environment interaction on yield, Fe and Zn content among locally cultivated common bean (*Phaseolus vulgaris* L.) germplasm

**DOI:** 10.3389/fpls.2026.1719270

**Published:** 2026-02-20

**Authors:** Eric Nduwarugira, Susan Nchimbi-Msolla, Paul M. Kusolwa, Teshale Assefa, Clare Mugisha Mukankusi, Jean Claude Rubyogo

**Affiliations:** 1 Department of Research, Institut des Sciences Agronomiques du Burundi (ISABU), Bujumbura, Burundi; 2 Department of Crop Science and Horticulture, Sokoine University of Agriculture (SUA), Morogoro, Tanzania; 3 Alliance of Bioversity (ABC) and International Center for Tropical Agriculture (CIAT) Arusha, c/o Selian Agricultural Research Institute, Arusha, Tanzania; 4 Alliance of Bioversity (ABC) and International Center for Tropical Agriculture (CIAT) Uganda, c/o National Agricultural Research Organisation (NARO) Kawanda Agricultural Research Institute, Kampala, Uganda; 5 Alliance of Bioversity (ABC) and International Center for Tropical Agriculture (CIAT) Kenya, c/o International Centre of Insect Physiology and Ecology (ICIPE), Nairobi, Kenya

**Keywords:** ammi, common bean, GxE interaction, heritability, micronutrients

## Abstract

**Introduction:**

Common bean (*Phaseolus vulgaris* L.) is a key source of dietary protein and micronutrients, in low- and middle-income countries. Improving both yield and micronutrient density requires a clear understanding of genotype (G), environment (E), and genotype-by-environment interaction (GEI) effects to support nutrition-sensitive breeding strategies.

**Methods:**

This study assessed the effects of G, E, and GEI on grain yield and micronutrient concentration using the Additive Main Effects and Multiplicative Interaction (AMMI) model. A total of 83 bush and 84 climbing common bean genotypes were evaluated across three locations and two cropping seasons (2024A–2024B) in Burundi. AMMI and GGE biplot analyses were applied to examine genotype performance, stability, and adaptation across environments. Broad-sense heritability was estimated for yield and micronutrient traits, and a multi-trait selection index (MTSI) was used to identify superior genotypes combining agronomic and nutritional attributes.

**Results:**

Combined AMMI-analysis revealed highly significant (p <0.001) effects of genotype and environment on grain yield, iron (Fe), and zinc (Zn), together with significant GEI for most traits. Significant genetic variation was observed for Fe (52.86–76.5 ppm in bush beans; 53.38–59.7 ppm in climbing beans), Zn (≈17–23 ppm), and grain yield (≈950–2240 kg ha^-1^). Several genotypes surpassed check varieties for Fe and Zn, though enhanced micronutrient levels were not consistently associated with high yield. AMMI and GGE biplots identified both stable, high-performing genotypes and those with specific environmental adaptation. Broad-sense heritability was high for Fe and Zn (h^2^ = 0.68–0.82) but low for yield (h^2^ ≈ 0.30–0.33). The multi-trait selection index (MTSI) effectively identified genotypes combining good yield and micronutrient density.

**Discussion:**

These findings highlight the importance of integrated multi-environment evaluation and multi-trait selection for developing high-yielding, micronutrient-dense common bean varieties adapted to diverse agro-ecologies.

## 1 Introduction

Common bean (*Phaseolus vulgaris* L.) is a nutritionally vital legume, widely consumed across the globe, especially in low-income countries where it serves as both a staple food and a cash crop (Aziziaram et al., 2021; Tryphone and Nchimbi-Msolla, 2010; Long et al., 2020). Rich in protein, iron (Fe), and zinc (Zn), it plays a key role in addressing micronutrient deficiencies, particularly among populations reliant on starch-based diets with limited diversity (Urwat et al., 2021; Diaz et al., 2022). Its adaptability to drought, poor soils, and varied climates makes it well-suited for cultivation in resource-limited settings (Moatshe-Mashiqa et al., 2021). In sub-Saharan Africa and Latin America, common beans are the most important grain legume for human consumption (Rubyogo et al., 2021). In Burundi, where micronutrient malnutrition remains prevalent especially among women and children, biofortified high-Fe and Zn bean varieties are being promoted to help combat anemia and related health issues (Nchanji et al., 2023a). In response, the Government of Burundi has launched various initiatives, including micronutrient supplementation programs and the fortification of staple foods, to combat these deficiencies (Rubyogo et al., 2021). Biofortification, defined as the enhancement of the nutritional quality of food crops through breeding and agronomy, is recognized as a cost-effective and sustainable strategy to improve public health (Gelaw et al., 2023; Bouis and Saltzman, 2017). It is also serving as a viable nutritional option for individuals following vegetarian diets in developed nations (Katuuramu et al., 2021).

Iron and zinc are essential for human growth, immunity, and cognitive development. Although required in small amounts, both minerals are vital for proper human nutrition (Tryphone and Nchimbi-Msolla, 2010). Regular bean consumption has been associated with a lower risk of various diseases, including cardiovascular diseases, diabetes, and cancer (de Lima et al., 2021). However, Fe and Zn deficiencies remain the leading causes of global micronutrient malnutrition, affecting individuals across all age groups (Diaz et al., 2022; Huertas et al., 2023). Iron deficiency can lead to increased maternal and infant morbidity and mortality, reduced resistance to infections, and impaired mental and psychomotor development in children. Similarly, zinc deficiency can negatively impact growth, cognitive function, and immune response, while also contributing to pregnancy complications and restricted fetal development (Stevens et al., 2024). Iron and zinc are essential micronutrients for normal human growth and development, yet their intake is often insufficient in the diets of vulnerable populations (Huertas et al., 2023). Consuming biofortified beans significantly improves iron levels in the blood, addressing a critical nutritional gap. Iron plays a crucial role in oxygen transport, metabolic energy production, immune defense, and cognitive development in children (Bouis and Saltzman, 2017). Similarly, zinc is vital for a strong immune system, cell division and growth, wound healing, reproduction, and the senses of smell and taste (Darnton-hill et al., 2005).

According to Blair et al. (2010), common bean was domesticated independently in Mesoamerica and the Andes, leading to two major gene pools that have been central to genetic improvement efforts for enhancing seed micronutrient levels. The Mesoamerican gene pool, originating from Central America and Mexico, is characterized by small to medium-sized seeds, while the Andean gene pool, from South America, typically has larger seeds. Previous studies have shown that Fe and Zn content vary depending on the gene pool, with Andean genotypes generally exhibiting higher Fe and Mesoamerican genotypes showing higher Zn concentrations; (Katuuramu et al., 2021; Blair et al., 2010). The observed variability is driven by genetic and environmental factors such as soil fertility, nutrient uptake, storage, temperature, and food preparation methods (Katuuramu et al., 2021; Welch and Graham, 2002). As a result, biofortification efforts in Mesoamerican beans face the challenge of increasing Fe levels without compromising Zn content, making the genetic analysis of seed mineral traits in this gene pool a priority (Blair et al., 2009).

Knowledge of heritability is a prerequisite for formulating scientifically sound breeding strategies. Estimating the heritability of key traits, including micronutrient concentrations, is essential for guiding parent selection in future breeding programs. The expression of maternal effects on iron and zinc concentrations in common bean seeds has been shown to depend on the gene pool under evaluation as well as the specific hybrid combinations tested (Magomere et al., 2024). Consequently, the development of mineral-dense genotypes through well-planned breeding programs requires comprehensive information on the extent of genetic variation and the heritability of economically important traits present in the target population (Govindaraj et al., 2011). The presence of sufficient genetic variation for micronutrient density is particularly critical in determining the feasibility of achieving substantial genetic gains through conventional breeding approaches (Graham et al., 1999). While narrow-sense heritability is useful for predicting response to selection, broad-sense heritability reflects the overall genetic control of a trait. Both measures are important to plant breeders, as the effectiveness of selection ultimately depends on the proportion of additive genetic variance relative to total phenotypic variance (Fernandez and Miller, 1985).

Despite global advancements in breeding and the release of improved high-Fe and high-Zn bean varieties in Burundi, the micronutrient composition of local landraces and their genotype-by-environment interactions remain inadequately studied. This knowledge gap limits the identification of nutrient-dense genotypes suitable for breeding, thus slowing efforts toward nutritional improvement. Although common beans are naturally rich in iron (Fe) and zinc (Zn), their micronutrient concentrations vary significantly due to genetic and environmental factors (Welch and Graham, 2002; Katuuramu et al., 2021). In common bean landraces, Fe ranges from 8.9 to over 100 mg kg^−1^ and Zn from 20 to 59 mg kg^−1^ (Talukder et al., 2010; Bulyaba et al., 2020). The average Fe content in common beans is estimated to be approximately 50 mg kg^−1^ (Glahn et al., 2020). Several studies have documented the genetic variation in these traits (Mukamuhirwa and Rurangwa, 2018; Katuuramu et al., 2021; Araújo et al., 2022). However, the effect of the environmental factors on common bean yield and grain mineral composition remains poorly understood as do the nutritional implications of these variations for human health (Bulyaba et al., 2020), especially in the context of Burundi. In soils with insufficient nutrients, plants may struggle to store adequate nutrients in their seeds (Graham et al., 2001).

Understanding the genetic and environmental factors affecting Fe and Zn accumulation in common beans is critical for the development of nutrient-rich varieties. Variations in yield, seed nutritional content, anti-nutritional compounds, and cooking qualities in common beans are influenced by both genotype and environmental factors, especially soil properties (Bulyaba et al., 2020; Hamabwe et al., 2024). Consequently, a crop’s ability to acquire and accumulate soil nutrients in its edible parts is shaped by the interaction between genotype and environment (Bulyaba et al., 2020). Successful breeding requires evaluating genotypes across multiple environments to select stable and high-performing landraces (Purchase et al., 2000). G × E interactions can slow down breeding progress because a genotype with good performance in one environment may perform poorly in another (Hamza et al., 2023). Environmental factors such as soil type, temperature, humidity, pests, and management practices also affect yield and mineral composition (Akcura and Kaya, 2008; Tryphone and Nchimbi-Msolla, 2010). Therefore, assessing these interactions is essential to guide breeding programs aimed at developing and recommending adaptable, nutrient-rich genotypes (Katuuramu et al., 2021; Araújo et al., 2022).

Recognizing these interactions is crucial for plant breeders to effectively develop, select, and recommend genotypes adapted to diverse environmental conditions (Tryphone and Nchimbi-Msolla, 2010). This study aimed to support breeding and food security in East Africa, with a particular focus on Burundi, by evaluating the performance and nutritional quality of common bean genotypes across diverse environments. Specifically, the study intended to evaluate the performance and stability of common bean genotypes for yield, Fe and Zn, including genotype × environment interactions, and to assess genetic potential and trait relationships through correlation analysis and heritability estimation. By achieving these objectives, the findings are expected to guide the development of common beans with improved nutritional quality and agronomic performance, adapted to diverse agro-ecological conditions.

## 2 Materials and methods

### 2.1 Plant materials

**Figure 1:**
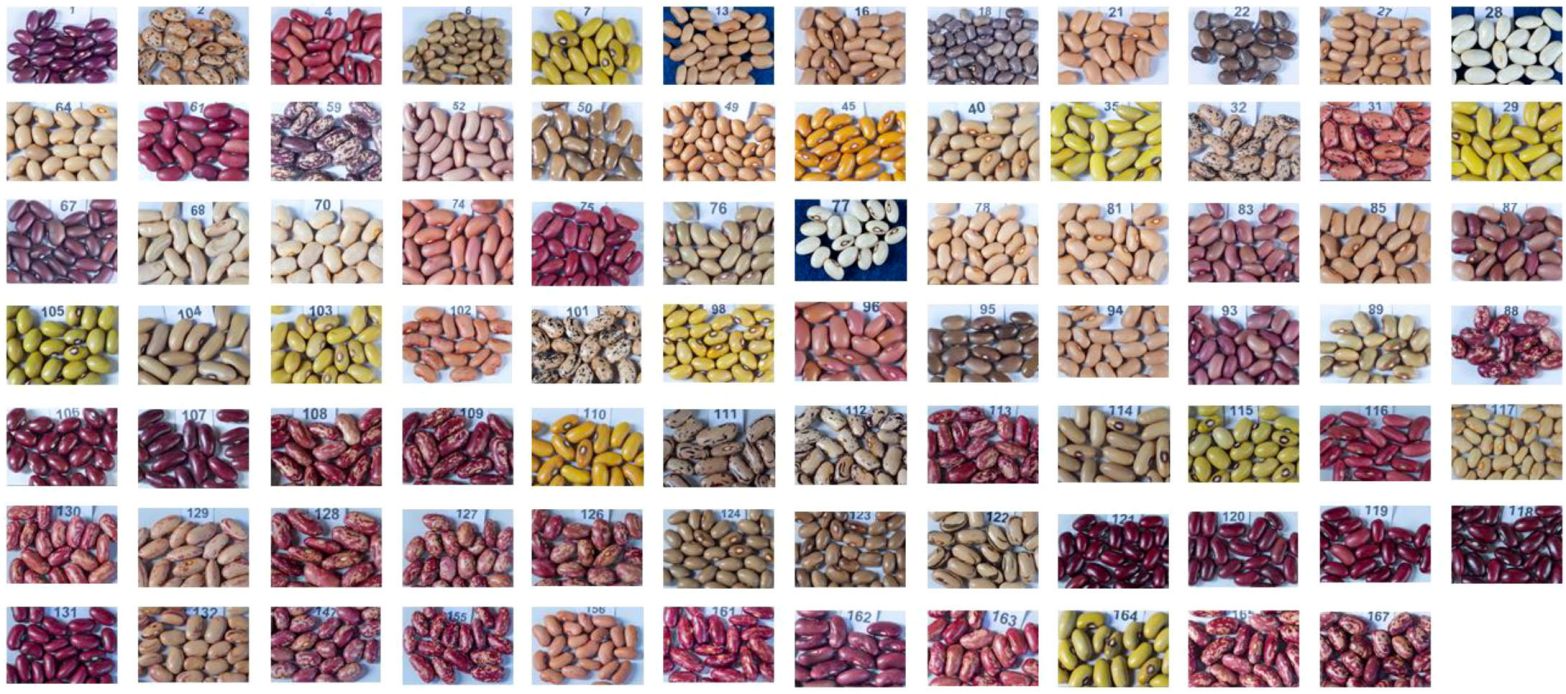
Bush bean genotypes used in the experiment.

**Figure 2:**
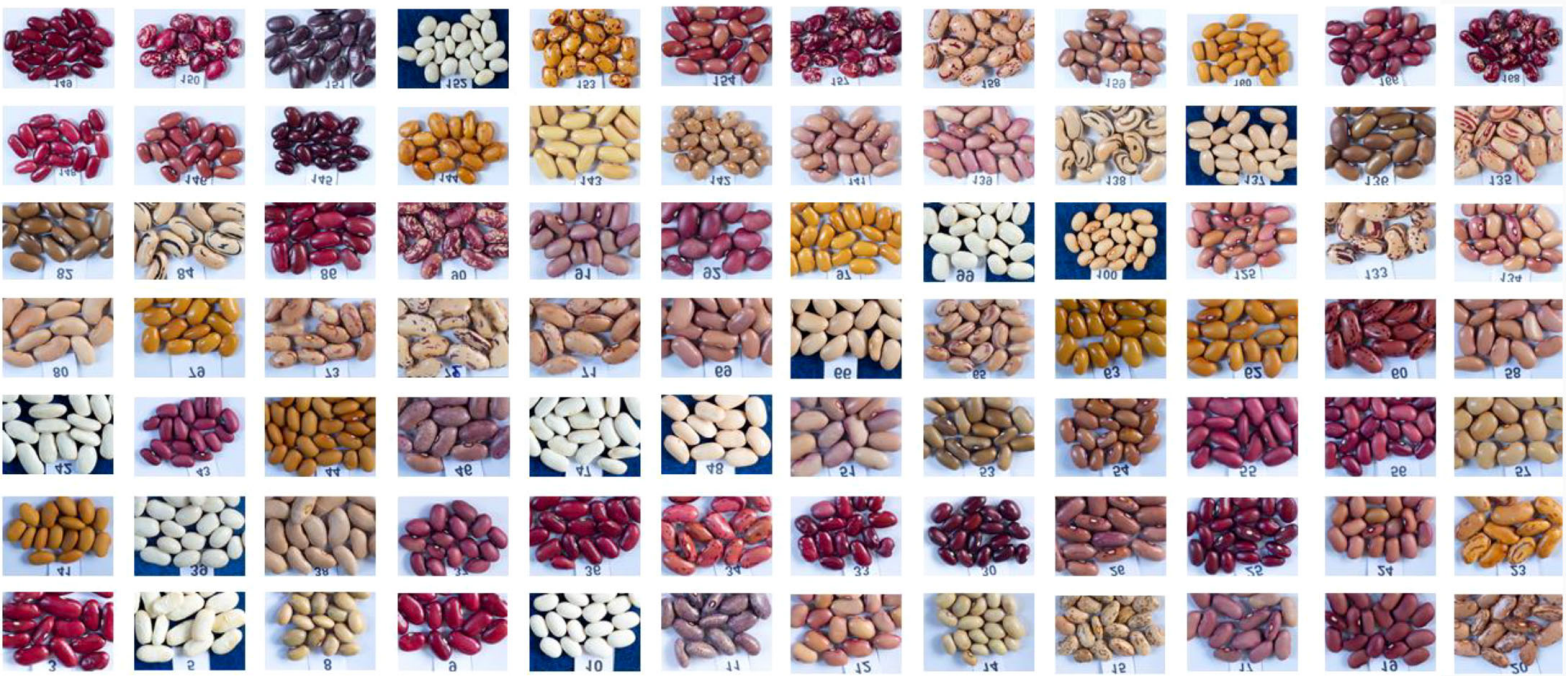
Climbing bean genotypes used in the experiment.

The study evaluated 84 climbing and 83 bush (determinate and indeterminate) bean genotypes, including 43 bush landraces, 40 improved bush beans, 23 improved climbing beans, and 61 climbing landraces. Among these, 54 improved varieties were released between 1990 and 2024, 11 were advanced breeding lines, and 102 landraces were sourced from Burundi’s National Gene Bank at ISABU. The germplasm includes representatives of both the Andean and Mesoamerican gene pools, reflected in its broad variation in seed size, ranging from small to large grains. This diverse germplasm represents most beans growing in Burundi’s for food supply and market, varying in seed shape, seed size, and seed color (**Figures 1**, **2**). In all trials and locations, MAC44 (Fe = 66 ppm, Zn = 28 ppm) and RWR2245 (Fe = 60 ppm, Zn = 30 ppm) were used as regional checks for biofortified bush and climbing bean varieties in Burundi, respectively, serving as reference standards for evaluating other genotypes (Nchanji et al., 2023b). Additionally, RWR2245 was the check for high Fe and Zn in bush beans, while IZO2015110 and G13607 were used as checks for low Fe and Zn in bush and climbing beans, respectively.

### 2.2 Description of experimental sites

The study was carried out at three ISABU research stations—Bukemba, Karusi, and Murongwe—across two cropping seasons: 2024A (Oct 2023–Jan 2024) and 2024B (Mar–Jun 2024). Bukemba, located in the low-altitude Moso region (latitude 4.00197°S, longitude 30.04211°E, altitude 1248 masl), records 1395 mm of rainfall annually and temperatures between 16 °C and 30 °C (**Table 1**). Soil analysis showed high Fe, Ca, K, and Mg, but low N, P, and Zn, with a moderately suitable pH for bean production (**Table 2**). Karusi, situated in the humid Kirimiro plateau (latitude 3.08943°S, longitude 30.17186°E, altitude 1587 masl), experiences 1422 mm of rainfall and temperatures ranging from 13 °C to 27 °C (**Table 1**). Its soil had high Fe, moderate K, N, and Mg, but low Ca, P, and Zn, and was slightly acidic—also moderately suitable for bean cultivation (**Table 2**). The Murongwe Innovation Center, located in the Kirimiro region (latitude 3.1924°S, longitude 29.89594°E, altitude 1531 masl), receives an average annual rainfall of 1408 mm with temperatures ranging from 16 °C to 28 °C (**Table 1**). Soil analysis revealed very high Fe concentrations, moderate levels of Ca, K, and Mg, but deficiencies in P, N, organic carbon, and Zn. The soil was slightly acidic, making it less suitable for bean cultivation (**Table 2**). Due to the risk of excessive rainfall and low temperatures, high-altitude sites were excluded to avoid damage to determinate bush beans. To improve soil fertility, organic matter was applied to supplement key deficient nutrients, including N, P, K, Ca, and Mg (**Table 3**).

**Table 1:** Agro-ecological description and climatic conditions during the growing cycle of the experimental sites.

Experimental site	Bukemba		Murongwe		Karusi	
Cropping season	2024A	2024B	2024A	2024B	2024A	2024B
altitude (m.a.s.l)	1200m	1200m	1497m	1508m	1597m	1594m
Longitude	30^0^4’47’’E	30^0^4’51’’E	29^0^53’56’’E	29^0^53’55’’E	30^0^10’20’’E	30^0^10’15’’E
Latitude	3^0^50’50S	3^0^59’56’’S	3^0^11’40’’S	3^0^11’44’’S	3^0^5’21’’S	3^0^5’23’’S
Min Temp	17.56167	16.47277	15.57597	14.90362	13.59027	13.33446
Max Temp	28.77949	29.63559	27.46882	28.46306	26.15815	27.29054
Mean Temp	23.2109	23.05418	21.54255	21.68334	19.87421	20.3125
Mean rainfall mm/season	265.425	173.25	251.475	190.6	237.525	207.95
Min Rel Hum	58.37688	52.72419	57.69301	52.97083	57.00914	53.21747
Max Rel Hum	95.04624	94.07204	95.84987	95.13441	96.65349	96.19677
Mean Rel Hum	76.71156	73.39812	76.77144	74.05262	76.83132	74.70712

Temperature and rainfall data were obtained from the Meteorological Agency of Burundi (IGEBU), Gitega office.

**Table 2:** Soil status of the experimental sites analyzed at ISABU soil lab.

Soil components	Bukemba			Murongwe			Karusi		
	2024A	2024B	Rating	2024A	2024B	Rating	2024A	2024B	Rating
pH in water	6.11	6.13	M	5.33	5.66	VSA	5.02	5.02	VSA
pH KCl	4.88	4.82	MA	4.31	4.61	VSA	4.27	4.33	VSA
Organic Carbon (%)	1.24	1.23	VL	1.62	1.47	VL	3.22	3.14	M
Total N (%)	0.196	0.198	Low	0.257	0.226	M	0.316	0.314	M
CEC (meq/100g)	21.5	19.9	M	15.9	15.8	Low	19	20.3	M
P (mg/kg)	≤2	≤2	Low	1.62	6.47	Low	≤2	≤2	Low
E. Ca (meq/100g)	9.91	10.5	High	5.01	6.38	M	1.4	1.5	Low
E. Mg (meq/100g)	5.8	5.33	High	1.71	1.89	M	1.46	1.15	M
E. K (meq/100g)	0.271	0.38	VH	0.072	0.138	M	0.209	0.253	M
E. Na (meq/100g)	≤0.05	0.02	Low	0.005	≤0.05	Low	0.008	0.006	Low
Fe (mg/kg)	53.7	65.9	High	64.1	58.9	High	118.6	88.1	High
Zn (mg/kg)	1.68	1.39	VL	2.12	1.83	VL	0.85	0.909	VL
TEA (meq/100g)	0.094	0.066	Low	0.513	0.151	Low	1.64	1.7	Low
Soil type	SCL	SCL		SL	SL		SL	SL	

VSA, Very Strong acidity; MA, moderately acidity; M, Medium; VL, Very low; VH, Very high; SCL, Sandy clay loam; SL, Silt loam; TEA, Total exchangeable acidity; E., Exchangeable; N: high = > 0.50, medium = 0.25-50; P: high => 40, medium = 20-40; K: high = 0.35, medium = 0.15 - 0.35; Ca: high = > 6.0, medium = 3.0-6.0; Mg: high => 2.5, medium = 1.5 - 2.5. (According to CSRO (2021).

**Table 3:** Organic manure status used in the experimental sites analyzed at ISABU soil lab.

Organic manure components	Bukemba		Murongwe		Karusi	
	2024A	2024B	2024A	2024B	2024A	2024B
pH in water	8.87	8.5	8.52	7.44	7.59	8.64
Organic Carbon (%)	14.3	12.5	10.9	15.5	17.5	9.62
Total N (%)	1.57	1.26	1.16	1.49	1.62	1.09
Total P (%)	1.17	0.591	0.58	0.362	0.542	0.551
Total K (%)	2.66	1.01	1.63	0.973	1.84	1.98
Ca (%)	3.32	0.849	0.956	0.555	0.797	0.902
Mg (%)	1.15	0.555	0.708	0.345	0.618	0.667

### 2.3 Experimental Design and trial establishment

The growing experiments for both bush and climbing genotypes were conducted using an unbalanced lattice design (Randomized Incomplete Block Design) with three replicates during both the 2024A and 2024B cropping seasons.

Each genotype was planted on a 3 m² plot with four 2 m long rows, spaced of 0.5 m between rows, and plants spaced 0.10 m within row. Recommended agronomic practices, such as applying 5–10 t/ha of organic manure and 150 kg/ha of organo-mineral fertilizer (FOMI Imbura N-P2O5-K2O-CaO-MgO), were followed at all locations. Soil samples were collected from each site at a 0–20 cm depth before applying organic manure and analyzed at the ISABU soil lab for physical and chemical properties, including total N, pH, organic matter, exchangeable bases, and extractable P, Zn, and Fe. Organic manure, applied uniformly across all sites regardless of soil results, was also analyzed for N, P, and K content. Meteorological data (temperature, rainfall, and humidity) were recorded throughout both seasons. Trials were rain-fed, and weeds were managed manually.

### 2.4 Seed iron (Fe) and zinc (Zn) analysis

In each trial, the sampling protocol established by (Stangoulis and Sison, 2008) was followed. At physiological maturity, fifteen well-filled pods were randomly sampled from each plot, cleaned by rubbing between cotton cloths to reduce contamination. The samples were then oven-dried at 60 °C for at least 12 hours, hand-threshed, and stored in labeled paper bags. Samples were milled and cleaned between runs, then analyzed for Fe and Zn at CIAT Kawanda using an Energy Dispersive X-ray Fluorescence (EDXRF) analyzer (Stangoulis and Sison, 2008).

The powdered samples were transferred into sample cups for XRF analysis. Fe and Zn concentrations were determined by scanning each sample for 60 seconds while rotating the cup. Standard samples were run daily for calibration, with MIB465 (Fe=81.2–84.1 ppm, Zn=22.5–26.26 ppm) and DOR500 (Fe=48.9–51.7 ppm, Zn=16.69–18.1 ppm) used as the high and low Fe checks, respectively.

### 2.5 Data analysis

Phenotypic data on grain iron (Fe) and zinc (Zn) concentrations and seed yield (kgha^-1^) were analyzed using R statistical software version 3.4.1. To assess significant differences in mineral concentrations and yield among common bean genotypes, To assess significant differences among common bean genotypes, a separate analysis of variance (ANOVA) was performed for each growth habit (bush and climbing) to account for inherent differences in growth type and performance. The ANOVA model included the effects of genotype, location, season, and their interactions, with replications considered within each location–season combination. Genotype and location effects were treated as fixed factors, while season, interaction terms, and residuals were considered random. This approach allowed for robust evaluation of genotype performance and environmental influence on yield and micronutrient concentration. Mean comparisons for grain yield, Fe, and Zn concentrations were conducted using Tukey’s Honest Significant Difference (HSD) test at a 5% level of significance. All ANOVA and subsequent mean separation analyses were performed using the agricolae R package (Olivoto and Lúcio, 2020).

#### 2.5.1 Additive main effects and multiplicative interaction model

The AMMI model was used to identify genotypes adapted to different environments by combining analysis of variance (ANOVA) and principal component axis (PCA). This model partitions the sources of variability in genotype-by-environment interaction (GEI) through PCA (Marjanović-Jeromela et al., 2011). The AMMI analysis, including the estimation of principal components and biplot visualization, was performed using the metan R package (Olivoto and Lúcio, 2020), which provides tools for multi-environment trial analysis in R. GEI was modeled according to the AMMI Equation 1.

#### 2.5.2 AMMI stability value analysis

AMMI Stability Value (ASV) was used to identify common bean genotypes with stable and high yields, as well as high Fe and Zn content. Since AMMI analysis does not directly measure stability, Purchase et al. (2000) recommended the use of ASV to rank genotypes based on yield stability and Fe/Zn content. A genotype’s stability is assessed through the ASV, where lower values correspond to greater stability. The ASV was calculated using weighted IPCA1 and IPCA2 scores, with the Equation 2.

#### 2.5.3 Mean performance and stability of multiple traits

Multi-trait selection index (MTSI) was performed to identify superior common bean genotypes based on grain yield, iron (Fe), and zinc (Zn) content following the approach described by (Olivoto et al., 2019). MTSI integrates both genotype mean performance and stability across multiple environments into a single, interpretable index. Stability for each trait is first assessed commonly using the weighted average of absolute scores from a biplot (WAASB), derived from the singular value decomposition of genotype environment BLUPs allowing both performance and genotype-by-environment interaction to be expressed on a common scale. Lower MTSI values indicate genotypes with high average performance and consistent behavior across tested environments, bringing them closer to an “ideal type.” The function mtsi() of the R package metan version 4.3.3. (Olivoto and Lúcio, 2020) was used to compute the index.

The Equation 3 was used to rescale traits. Factor analysis was then applied to the rescaled data to account for trait correlations and reduce dimensionality, following the Equation 4. An ideotype was defined based on desired trait values, and the MTSI for each genotype was calculated as the Euclidean distance between genotype and ideotype scores in factor space (Equation 5). $\gamma_{ij}\gamma_{j}$

#### 2.5.4 Genotype and genotype by environment – biplot analysis

The GGE biplot methodology was used to analyze multi-location genotype data, assessing the stability of yield (kgha^-1^), Fe and Zn and identifying superior genotypes using R 3.4.1 software. The GGE biplot analysis also generated graphs for: (i) comparing environments to an ideal environment, (ii) identifying the “which-won-where” pattern, and (iii) visualizing environment vectors.

The angles between these vectors were used to evaluate the correlations (similarities or dissimilarities) between pairs of environments (Yan and Kang, 2002).

#### 2.5.5 Broad sense heritability

For the combined analysis across environments, broad-sense heritability was estimated using R software (version 3.4.1) following the Equation 6.


$$\sigma_{g}^{2}{\text{σ}}_{g*l}^{2}\sigma_{e}^{2}\sigma_{g}^{2}\sigma_{g\times e}^{2}\sigma_{e}^{2}n_{\text{env}}n_{\text{reps}}Y_{ijk}=\;A=\mu +\;E_{j\;}+GE_{ij}R_{k\;}E_{J}+\;\epsilon_{ijk}Y_{ijk}\mu G_{i}i^{th}E_{j\;}j^{th}GE_{ij}R_{k\;}E_{J}\epsilon_{ijk}$$

## 3 Results and discussion

### 3.1 Results

#### 3.1.1 ANOVA of main effects and multiplicative interaction for Fe, Zn and yield (kgha^-1^) in bush and climbing bean genotypes

Common bean (*Phaseolus vulgaris* L.) is a key source of dietary protein and micronutrients in low- and middle-income countries. To support nutrition-sensitive breeding, this study evaluated the effects of genotype (G), environment (E), and genotype-by-environment interaction (GEI) on grain yield and micronutrient content using the AMMI model, testing 83 bush and 84 climbing genotypes across three locations and two growing seasons (2024A–2024B) in Burundi.

Combined AMMI-analysis revealed highly significant (p < 0.001) effects of genotype and environment on grain yield, iron (Fe), and zinc (Zn), together with significant GEI for most traits, highlighting the influence of both genetic variation and environmental heterogeneity on genotype performance (**Table 4**). Significant variation was observed among genotypes for Fe concentration (52.86–76.5 ppm in bush beans; 53.38–59.7 ppm in climbing beans), Zn concentration (≈17–23 ppm), and grain yield (≈950–2240 kg ha^−1^). Several genotypes outperformed check varieties for Fe and Zn, although enhanced micronutrient levels were not consistently associated with high grain yield.

**Table 4:** Summary of combined analyses of variance from AMMI model for Fe, Zn and yield (kgha^-1^) in bush and climbing bean.

GH	Source	df	Fe (2024A)	Zn (2024A)	GYD_kgha^−1^ (2024A)	Fe (2024B)	Zn (2024B)	GYD_kgha^−1^ (2024B)	Fe (Combined)	Zn (Combined)	GYD_kgha^−1^ (Combined)
Bush	ENV	2	460.7*	14 25.0680	8298 409	313.667	97.628*	121498629**	554.69**	47.674*	68137351**
	Block	6	68.39	86 14.4774***	5348 261***	62.895*	15.378***	5755846***	31.88	5.134	4886256***
	Gen	82	462.9***	85 27.0957***	4569 118***	207.685***	24.7***	1298324***	479.35***	43.107***	1973091***
	Env*Gen	164	43.97*	82 2.7428	4821 34***	41.152***	6.204***	518807***	45.81	5.061**	416036
	PCA1	83	50.04561*	3.321742*	488529.6***	46.9925***	9.067420***	672492.7***	27.22126	3.605234	263175.7
	PCA2	81	37.74419	2.14956	204035.4	35.16691*	3.269569	361325.9	18.4809	1.429178	151498
	Residuals	492	35.27	59 2.3854	3201 18	26.564	3.881	319935	47.19	3.83	826410
Climbing	ENV	2	556.43	70.916*	33 266544	2228.07**	307.216**	59980022*	2347.9**	47.674*	68137351**
	Block	6	210.48***	11.372***	1355410***	134.41***	14.146**	6382987***	129.43***	5.134	4886256***
	Gen	83	226.87***	20.01***	9115175***	172.8***	15.828***	1532804***	326.86***	43.107***	1973091***
	Env*Gen	166	28.91	2.908	215603***	43.53***	7.369***	737219***	38.44	5.061**	416036
	PCA1	84	33.39088	3.282503	780232.9***	46.92274***	8.226841***	883810.5***	20.25672	3.605234	263175.7
	PCA2	82	24.32011	2.523736	335032.4	40.04697**	6.490597*	587052*	18.15731	1.429178	151498
	Residuals	498	26.6	2.638	993026	26.77	3.932	44760	32.64	3.83	826410

Key: ***p<-0.001, **p<-0.01 and *p<-0.05, Fe, Iron; Zn, Zinc; GYD_kg, grain yield in kg; df, degree of freedom; ENV, Environment; GEN, Genotype; PCA, Principal Component Axis; df, degree of freedom; Fe, Iron; Zn, Zinc; GYD_kgha^-1^, Grain yield in kg per hectar.

AMMI and GGE biplot analyses identified stable, high-performing genotypes across environments. Stability analyses using IPCA scores and AMMI values distinguished stable genotypes from those with environment-specific adaptation, while GGE biplots highlighted discriminative environments and broadly or specifically adapted genotypes. Broad-sense heritability was high for Fe and Zn (h² = 0.68–0.82) but low for yield (h² ≈ 0.30–0.33). The multi-trait selection index (MTSI) effectively identified genotypes combining good yield and micronutrient density. Overall, these findings support multi-environment, integrated selection for breeding high-yielding, nutrient-rich common beans adapted to diverse agro-ecologies.

#### 3.1.2 AMMI analysis for mean performance and stability of bush and climbing bean genotypes

**Table 5** presents the top 15 bush and climbing bean genotypes based on mean performance for Fe, Zn, and grain yield, along with IPCA scores and AMMI Stability Values (ASV). Complete results are provided in Appendices A3, A4.

**Table 5:** Top 15 performing bush and climbing bean genotypes based on high Fe, Zn, yield, ASV, and IPCA values.

Bush							Climber						
genotypes	Fe	Zn	Yield (kgha^-1^)	IPCA1	IPCA2	ASV	Genotypes	Fe (ppm)	Zn (ppm)	Yield (kg/ha)	IPCA1	IPCA2	ASV
132. KWC51	76.5a	22.87ab	1116.89b	-3.66	-2.54	7	142. Nakaje	59.70a	21.39a-g	1852.06a-c	1.17	6.27	6.89
167. DAB576	68.82ab	23.10a	1519.06ab	2.92	0.94	5.28	141. Murengeti	59.26ab	21.01a-i	1235.83a-c	-6.78	-5.03	17.44
68. NUS17	59.58bc	20.98a-f	895b	3.4	-1.24	6.18	90. Gasimbo	55.85a-c	21.83a-d	2241.89a-c	2.34	7.65	9.58
27. Sinamakamwe	57.43b-d	17.42g-n	1730ab	3.29	-7.31	9.37	159. Mbagarumbise	55.50a-d	20.47a-k	1995.94a-c	-2.98	-6.97	10.12
77. Ncocere	56.19c-e	19.69a-l	1160.67b	-0.32	-5.18	5.21	158. MAC52	55.49a-d	19.86a-l	1608.78a-c	0.96	0.78	2.48
165. Runyange2	54.94c-f	19.73a-l	1221.33b	4.58	-9.32	12.39	125. Pfahuntereye	55.06a-e	20.48a-j	1426.17a-c	2.46	-3.45	6.98
96. NUS31	54.47c-f	20.32a-j	947.17b	-2.4	-0.11	4.27	157. MAC13	55.02a-f	20.21a-l	1688.00a-c	-11.68	5.55	29.28
147. DAB570	54.2c-f	19.23d-l	1244.89b	-5.63	-8.72	13.28	168. Magorori	54.88a-g	18.06g-l	2107.22a-c	-20.37	2.94	50.22
74. NUS13	53.67c-g	22.42a-d	1292.61b	-0.02	-3.95	3.95	134. RWV1272	54.65a-g	22.64ab	1694.39a-c	-6.85	1.05	16.89
76. Agaharawe	53.65c-g	20.74a-h	1420.39ab	-2.82	1.19	5.16	152. VCB81013	54.27a-g	20.11a-l	1531.33a-c	-1.76	-4.74	6.42
95. Kiryinkumi	53.50c-g	20.84a-g	1712.17ab	-3.39	8.29	10.25	153. Bihogo	54.18a-h	22.44a-c	1141.72a-c	-1.09	-0.64	2.76
7. Kajemunkangara	53.37c-g	19.27d-l	1586.44ab	-5.22	1.19	9.37	62. Mbagara	53.99a-i	19.72a-l	1845.28a-c	3.67	-5.35	10.51
31. Kijumbura	53.21c-g	17.87f-n	860.44b	2.19	0.88	4	10. Runyenyeri	53.97a-i	19.74a-l	1229.28a-c	-4.31	-14.2	17.73
113. Kaneza	53.19c-g	19.13d-l	1604.33ab	-3.45	-1.1	6.24	8. Ndimirinkobe	53.82a-i	21.74a-e	1352.50a-c	2.99	-1.07	7.44
32. Vondoro	52.86c-h	19.89a-l	1343.06b	4.08	3.37	8	137. GSZ611	53.38a-j	20.21a-l	1733.39a-c	-10.48	1.61	25.85

For bush beans, genotype 132 exhibited the highest Fe content (76.5 ppm) with moderate Zn (22.87 ppm). Fe concentrations among the top 15 bush genotypes ranged from 52.86 to 76.5 ppm, while Zn concentrations ranged from 17.42 to 23.10 ppm. Thirteen bush bean genotypes (132, 167, 68, 27, 77, 165, 96, 147, 74, 76, 95, 7, and 31), excluding genotype 27 for Zn, surpassed the check (genotype 113) in Fe and Zn content. Genotype 27 recorded the highest mean yield (1730 kgha^−1^), while genotype 167 combined relatively high yield (1519.06 kgha^−1^) with favorable Fe and Zn concentrations. Genotype 96 had the lowest yield (947.17 kgha^−1^).

Stability analysis showed considerable variation in IPCA scores and ASV among bush bean genotypes. Genotype 132 had relatively low interaction effects, whereas genotype 165 exhibited the highest ASV. Several genotypes with lower ASV values displayed moderate yield and micronutrient concentration.

For climbing beans, Fe concentration among the top 15 genotypes ranged from 53.38 to 59.70 ppm, while Zn ranged from 18.06 to 22.64 ppm. Grain yield ranged from 1141.72 to 2241.89 kg/ha. Genotype 142 recorded the highest Fe concentration (59.70 ppm), whereas genotype 134 had the highest Zn concentration (22.64 ppm). The check (168) produced a grain yield of 2107.22 kg/ha. Ten genotypes (142, 141, 90, 159, 158, 125, 157, 134, 152, and 153) exceeded or equaled the check for Fe concentration, and fourteen genotypes exceeded the check for Zn. Genotype 141 showed high Fe and Zn but relatively low yield, while genotype 90 combined high yield with acceptable micronutrient levels. Genotype 168 expressed strong interaction effects as indicated by high IPCA1 (–20.37) and ASV (50.22) values, whereas genotype 142 was comparatively more stable.

#### 3.1.3 Multi-trait selection index (MTSI) for bush and climbing bean genotypes

The multi-trait selection index was applied to simultaneously evaluate grain yield, Fe, and Zn concentrations in bush and climbing bean genotypes (**Table 6**; Appendices A5, A6). In bush beans, genotypes 167 (DAB576) and 132 (KWC51) ranked highest, combining high Fe (68.8–76.5 mg/kg), competitive Zn (22.9–23.1 mg/kg), and acceptable grain yield. Genotypes such as 95 (Kiryinkumi), 93 (Tsindinzara), and 2 (Urubonobono) obtained high MTSI values due to balanced contributions of yield and micronutrients. Genotypes with very high yield but moderate micronutrient concentrations were ranked lower. In climbing beans, genotype 90 (Gasimbo) ranked first, integrating high grain yield (≈2242 kg/ha) with elevated Fe and Zn concentrations. Other genotypes including 142 (Nakaje), 159 (Mbagarumbise), and 134 (RWV1272) showed balanced multi-trait performance. Variation in ranking reflected differing trait combinations among genotypes.

**Table 6:** Top 15 high performing bush and climbing bean genotypes based on the multi-trait stability index (MTSI).

Rank	Genotype	GH	GYD_kg	Fe	Zn	GYD_r	Fe_r	Zn_r	MTSI
1	167.DAB576	Bush	1519	68.8	23.1	0.428	0.783	1	0.612
2	132.KWC51	Bush	1117	76.5	22.9	0.237	1	0.973	0.764
3	95.Kiryinkumi	Bush	1712	53.5	20.8	0.52	0.349	0.731	0.852
4	93.Tsindinzara	Bush	1790	52.8	19.9	0.558	0.33	0.619	0.888
5	29.Karabera	Bush	1578	52.1	20.6	0.456	0.31	0.701	0.928
6	67.Mufyiri	Bush	1432	52.7	21.5	0.387	0.327	0.814	0.93
7	76.Agaharawe	Bush	1420	53.7	20.7	0.381	0.353	0.719	0.938
8	74.Amasera	Bush	1293	53.7	22.4	0.32	0.354	0.919	0.941
9	2.Urubonobono	Bush	1885	49.2	20	0.603	0.228	0.632	0.943
10	89.Zimanumukwe	Bush	1623	52.4	19.8	0.478	0.317	0.601	0.948
11	156.Sinamino_b2	Bush	1659	52.1	19.6	0.495	0.31	0.579	0.953
12	50.Marasira	Bush	1666	50.2	20.3	0.498	0.255	0.666	0.958
13	49.Ubudida	Bush	1528	50.6	21	0.433	0.268	0.747	0.96
14	7.Kajemunkangara	Bush	1586	53.4	19.3	0.46	0.345	0.544	0.963
15	113.RWR2245	Bush	1604	53.2	19.1	0.469	0.34	0.527	0.97
1	90.Gasimbo	Climbing	2242	55.8	21.8	0.784	0.808	0.845	0.328
2	142.Nakaje	Climbing	1852	59.7	21.4	0.532	1	0.77	0.521
3	159.Mbagarumbise	Climbing	1996	55.5	20.5	0.625	0.79	0.613	0.578
4	134.RWV1272	Climbing	1694	54.7	22.6	0.43	0.748	0.982	0.623
5	19.Naruhengeri	Climbing	1728	53	22.7	0.452	0.665	1	0.643
6	55.Bwijurihe	Climbing	2013	51.8	20.6	0.636	0.605	0.628	0.653
7	138. Muhoro	Climbing	2114	48.6	21.4	0.701	0.444	0.767	0.672
8	145.NUV130	Climbing	2016	48.9	20.6	0.638	0.46	0.641	0.742
9	15.Amavondoro	Climbing	1872	52.4	20	0.545	0.634	0.529	0.75
10	62.Mbagara	Climbing	1845	54	19.7	0.528	0.715	0.486	0.754
11	157.MAC13	Climbing	1688	55	20.2	0.426	0.767	0.57	0.754
12	146.NUV91	Climbing	1754	51.7	20.6	0.469	0.603	0.634	0.758
13	24.Nokia_c1	Climbing	1661	51.3	21.4	0.409	0.583	0.769	0.76
14	137.GSZ611	Climbing	1733	53.4	20.2	0.455	0.685	0.57	0.762
15	97.Rusenyanzego	Climbing	2231	47.6	20.3	0.777	0.397	0.59	0.762

#### 3.1.4 Broad sense heritability

Broad-sense heritability (h²) estimates for Fe, Zn, and grain yield in bush and climbing beans across environments are presented in **Table 7**. In bush beans, heritability estimates were high for Fe (0.81) and Zn (0.82), but lower for grain yield (0.33). In climbing beans, Fe and Zn exhibited moderate to high heritability (0.76 and 0.68, respectively), while grain yield showed low heritability (0.30). Phenotypic variance was highest for grain yield in both growth habits, followed by Fe and Zn.

**Table 7:** Broad sense heritability analysis for both bush and climbing bean genotypes across locations and seasons.

	Bush bean genotypes			Climbing bean genotypes		
Parameter	Fe	Zn	GYD_kg	Fe	Zn	GYD_kg
Phenotypic variance	29.58	2.65	198,909.20	21.56	2.03	206,107.50
Heritability	0.81	0.82	0.33	0.76	0.68	0.3

#### 3.1.5 Correlation analysis between Fe, Zn and Yield (kgha-1) for bush and climbing bean genotypes across locations and seasons

**Figure 3:**
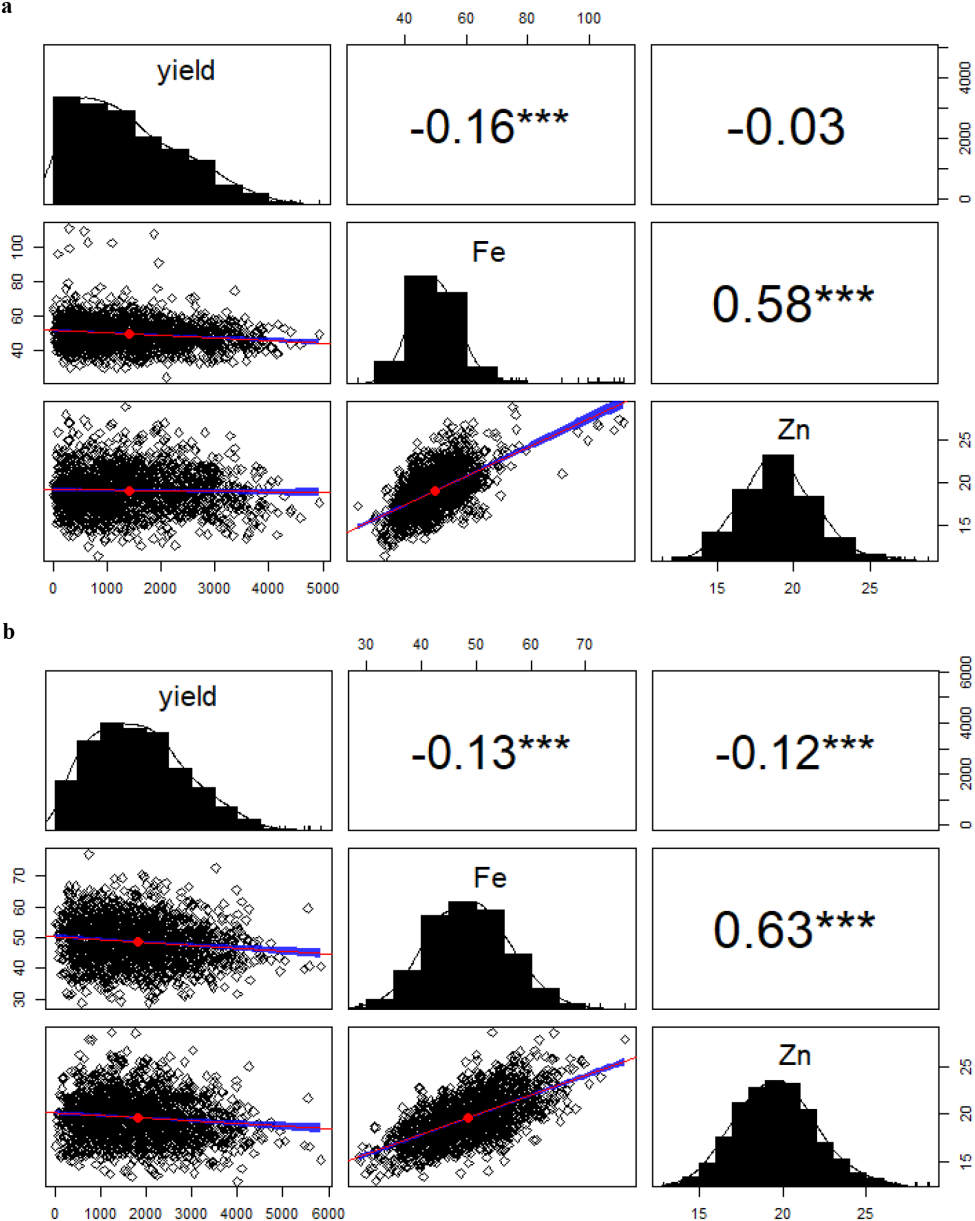
**(a)** Correlation analysis between Fe, Zn and Yield (kg/ha) for bush bean genotypes across locations and seasons. **(b)** Correlation analysis between Fe, Zn and Yield (GYD_kgha^-1^) for climbing bean genotypes across locations. ***: Highly significant.

**Figure 3a** shows correlations among yield, Fe, and Zn in bush beans. Yield was negatively correlated with Fe (-0.16***), while Zn showed a weak, non-significant correlation with yield. Fe and Zn exhibited a strong positive correlation (0.58***). Similarly, **Figure 3b** shows that in climbing beans, yield had weak but significant negative correlations with Fe and Zn, while Fe and Zn were strongly positively correlated.

#### 3.1.6 Genotype plus genotype-by-environment biplot for bush bean genotypes across environments

##### 3.1.6.1 GGE biplot for Fe in bush bean genotypes

**Figure 4:**
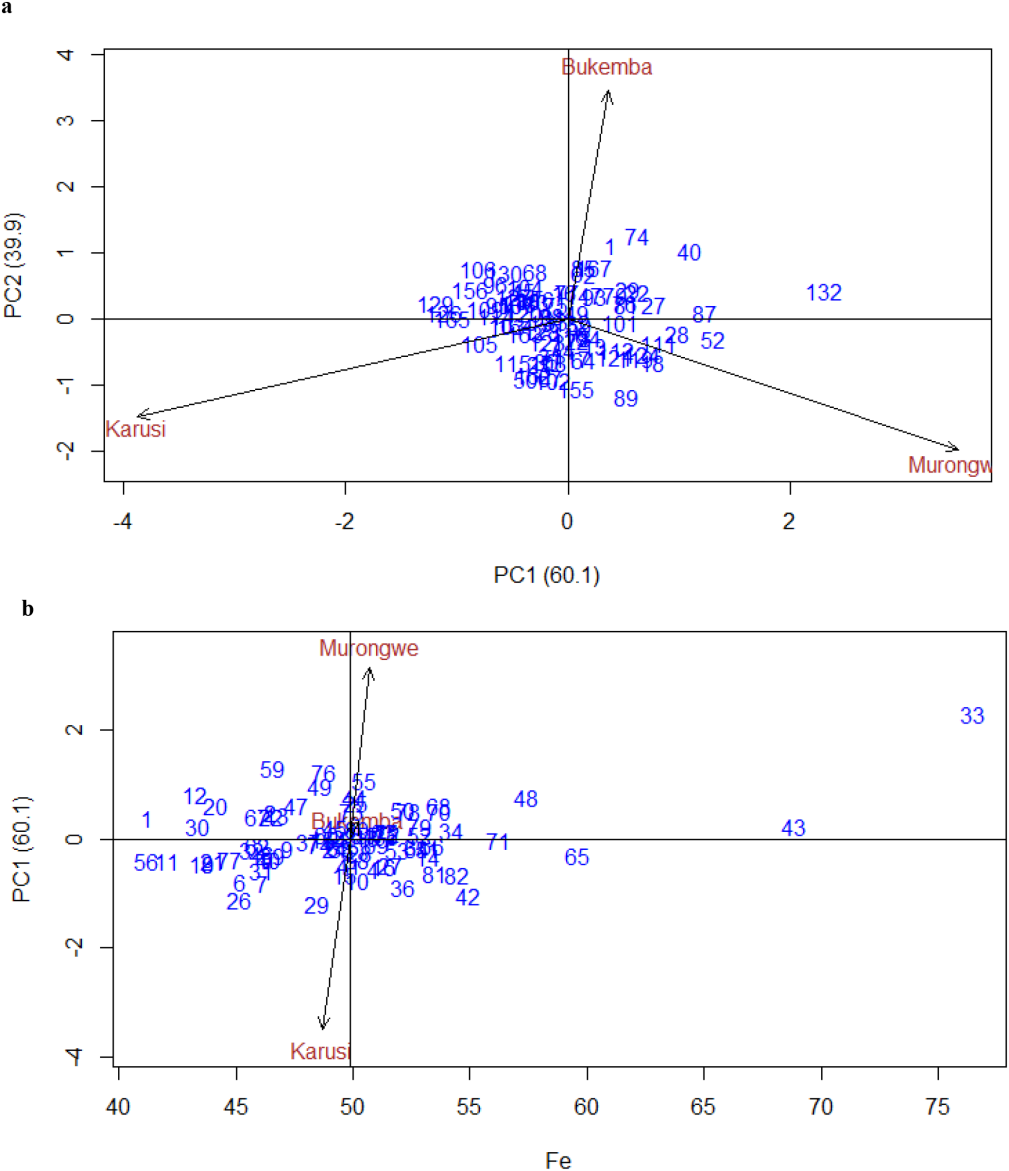
**(a)** GGE biplot for Fe PCA1 and PCA2 in bush bean genotypes across environments. **(b)** Biplot for Fe of the bush bean genotypes and environment IPCA score against means.

AMMI biplots (**Figures 4a, b**) illustrated genotype adaptability and stability. The **Figure 4a** provides an overview of the interrelationships between environments in relation to Fe content across genotypes. PC1 and PC2 explained 60.1% and 39.9% of the GEI variation, respectively, jointly accounting for 100% of the interaction due to the limited number of test environments, thereby allowing a complete representation of GEI patterns in the biplot. Because the analysis involved only three environments, these two components accounted for 100% of the GEI, allowing complete visualization of genotype–environment patterns in the biplot. Genotypes with positive PC1 (greater than zero) scores had above-average Fe content, while negative scores indicated lower Fe levels. Stability was associated with PC2 scores near zero. Genotypes near the IPCA origin showed minimal interaction effects, while those further away were more sensitive. The PCA1 vs. PCA2 plot revealed negative correlations among environments, indicated by angles exceeding 90°. **Figure 4b** presents the AMMI biplot for Fe content in bush bean genotypes and environment IPCA score against means. Most genotypes (e.g., 31, 34, 9, 71 bulked lines) were near the origin, indicating average stability but moderate Fe levels while some showed specific adaptation to environments: Genotype 33 exhibited the highest Fe content but low stability due to a high IPCA1 score. Murongwe favored high-Fe genotypes such as 33, 43, 66, 71 and 48 while Karusi (29, 26, 6, 56, 11, 36, 42) negatively affected Fe accumulation. For breeding programs targeting both high and stable Fe content, genotypes bulked near the origin such as 47, 34, 68, 81, 36 may be optimal.

##### 3.1.6.2 GGE biplot for Zn in bush bean genotypes

**Figure 5:**
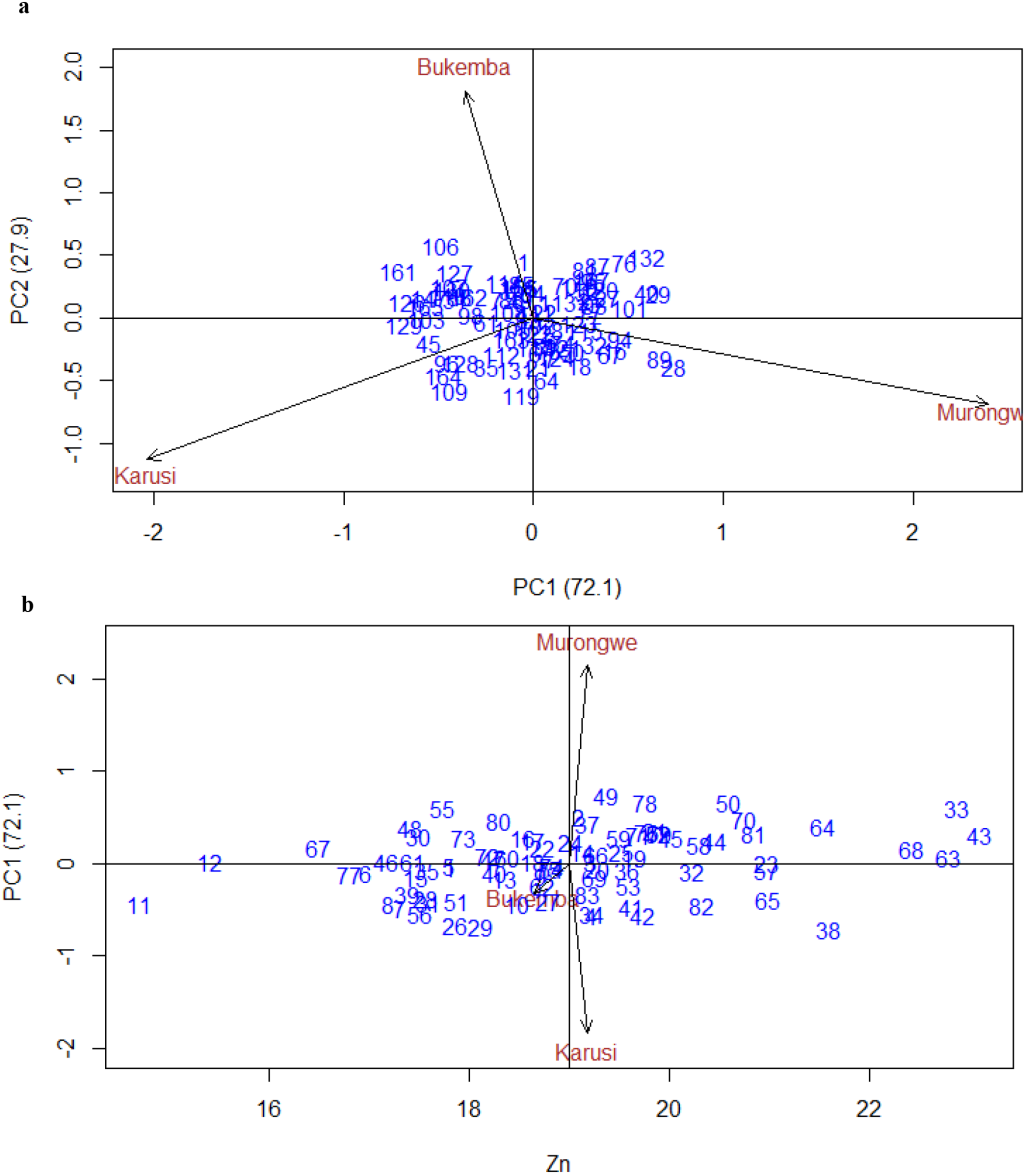
**(a)** GGE biplot for Zn_PCA1 vs PCA2 bush bean genotypes across environments. **(b)** Biplot for Zn of the bush bean genotypes and environment IPCA score against means.

**Figures 5a, b** show the GGE biplots for Zn concentration. PC1 explained 72.1% of the variation, and PC2 explained 27.9%. Murongwe and Karusi contributed strongly to G×E interaction, while Bukemba showed shorter vectors, indicating lower interaction effects and greater stability. Karusi and Murongwe had longer vectors than Bukemba, indicating a greater contribution to environmental variation, consistent with ANOVA results. The opposite directions of their vectors suggest a negative correlation, reflecting contrasting conditions for Zn accumulation. Murongwe and Bukemba were also negatively correlated, as shown by the obtuse angle between their arrows. In contrast, Bukemba and Karusi were positively correlated as indicated by the acute angle, implying similar genotype performance and that one environment could represent the other in selection.

Similarly, **Figure 5b** presents the interaction principal component axis (IPCA) scores plotted against the mean Zn concentration of bush bean genotypes across environments. Genotypes located near the origin (e.g., 55, 48, 70, 29, 30, 58, 4, 81, 26, and 51, including bulked genotypes) demonstrated stability across environments, though with relatively lower Zn levels, indicating general adaptability. In contrast, genotypes positioned farther from the biplot origin—such as 26, 29, 42, and 38 in Karusi, and 43, 63, 33, 68, 64, 49, 78, and 50 in Murongwe—exhibited stronger genotype × environment interactions and superior performance specific to their respective test sites. Murongwe had a high positive IPCA score, while Karusi had a high negative one. Bukemba, positioned near the origin with a short vector, showed minimal interaction, confirming its role as a stable and representative environment for Zn evaluation.

##### 3.1.6.3 GGE biplot for GYD_kgha^-1^ in bush bean genotypes

**Figure 6:**
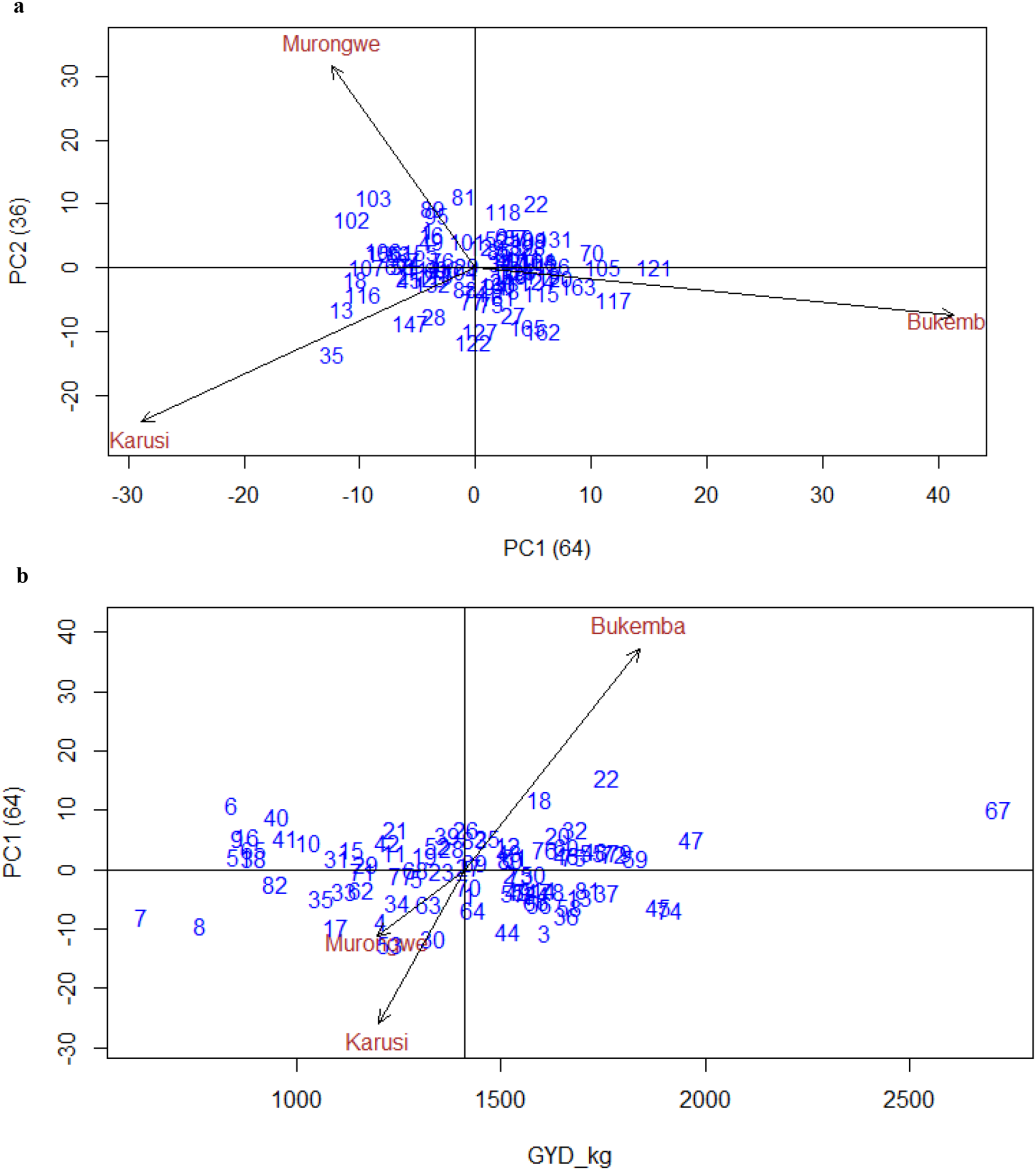
**(a)** GGE biplot for GYD_kgha^-1^_PCA1 vs PCA2 bush bean genotypes across environments. **(b)** Biplot for GYD_kgha^-1^ of the bush bean genotypes and environment IPCA score against means.

**Figure 6a** illustrates the performance and stability of bush bean genotypes for grain yield (GYD_kgha^−1^) across multiple environments. The first principal component (PC1) explained 64% of the variation, while the second (PC2) accounted for 36%, jointly capturing the entire genotype × environment interaction due to the limited number of test environments. As only three environments were included in the analysis, the G×E matrix was inherently restricted to two principal components; consequently, PC1 and PC2 together explained 100% of the interaction variation. The acute angle between Karusi and Murongwe suggests a positive correlation between these environments.

In addition, **Figure 6b** presents the yield performance of bush bean genotypes (GYD_kg/ha) across environments by plotting their mean yield (horizontal axis) against IPCA scores (vertical axis), which indicate genotype-environment interaction. Genotypes, particularly those clustered near the origin including bulked entries such as 15, 42, 21, 3, 7, and 66, exhibited average yield and broad stability, suggesting general adaptation. Conversely, genotypes with high IPCA scores such as 63, 64, 44, 3 (Karusi); 4, 17, 8, 34, 35, 62, 7 (Murongwe); and 18, 22, 47, 2, 67 (Bukemba) exhibited greater interaction effects and specific adaptation.

#### 3.1.7 Genotype plus genotype by environment biplot for climbing bean genotypes

##### 3.1.7.1 GGE biplot for Fe in climbing bean genotypes

**Figure 7:**
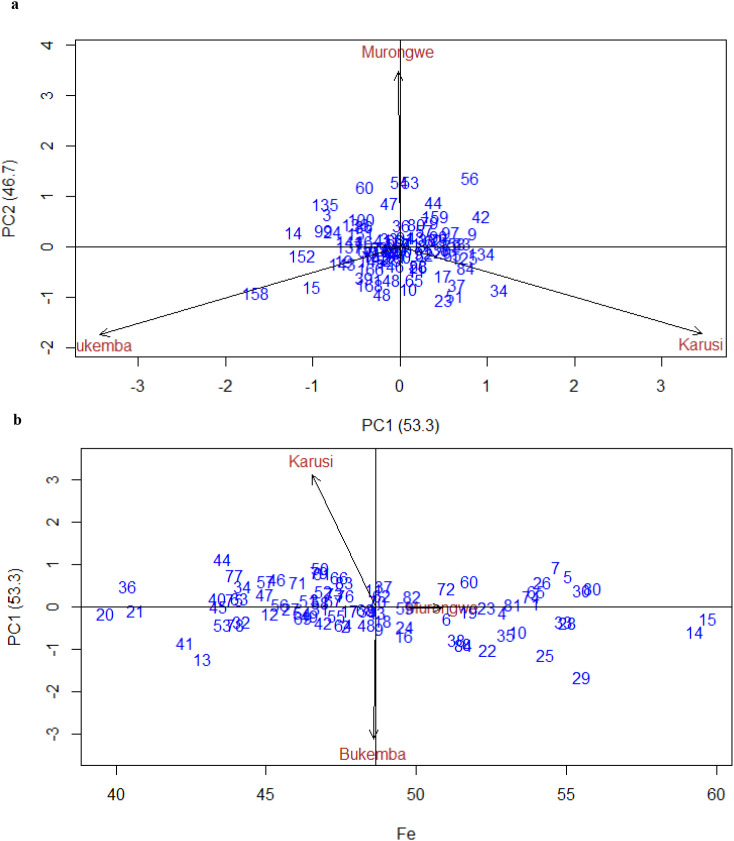
**(a)** GGE biplot for Fe PCA1 vs, PCA2 for climbing bean genotypes across environments. **(b)** Biplot for Fe of the climbing bean genotypes and environment IPCA score against means.

**Figures 7a, b** illustrate the GGE biplots for Fe concentration. In the **Figure 7a**, PC1 and PC2 explained 53.3% and 46.7% of the total variation, respectively. Bukemba and Karusi displayed strong interaction effects, while Murongwe was relatively stable. Negative correlations among Bukemba, Murongwe, and Karusi were indicated by the obtuse angles between their vectors. **Figure 7b** presents the AMMI biplot for Fe concentration in climbing bean genotypes across environments. Genotypes positioned near the origin such as 82, 72, 48, 24, 70, 66, and 71 exhibited minimal genotype-by-environment interaction, indicating stable performance across locations. In contrast, genotypes like 44 and 77 (Karusi), and 13, 41, 22, 25, and 29 (Bukemba) were located farther from the origin, reflecting strong interaction effects and environment-specific adaptation. Murongwe, with its short vector and central placement, showed limited interaction with genotypes, making it a stable and representative site for Fe evaluation.

##### 3.1.7.2 GGE biplot for Zn in climbing bean genotypes

**Figure 8:**
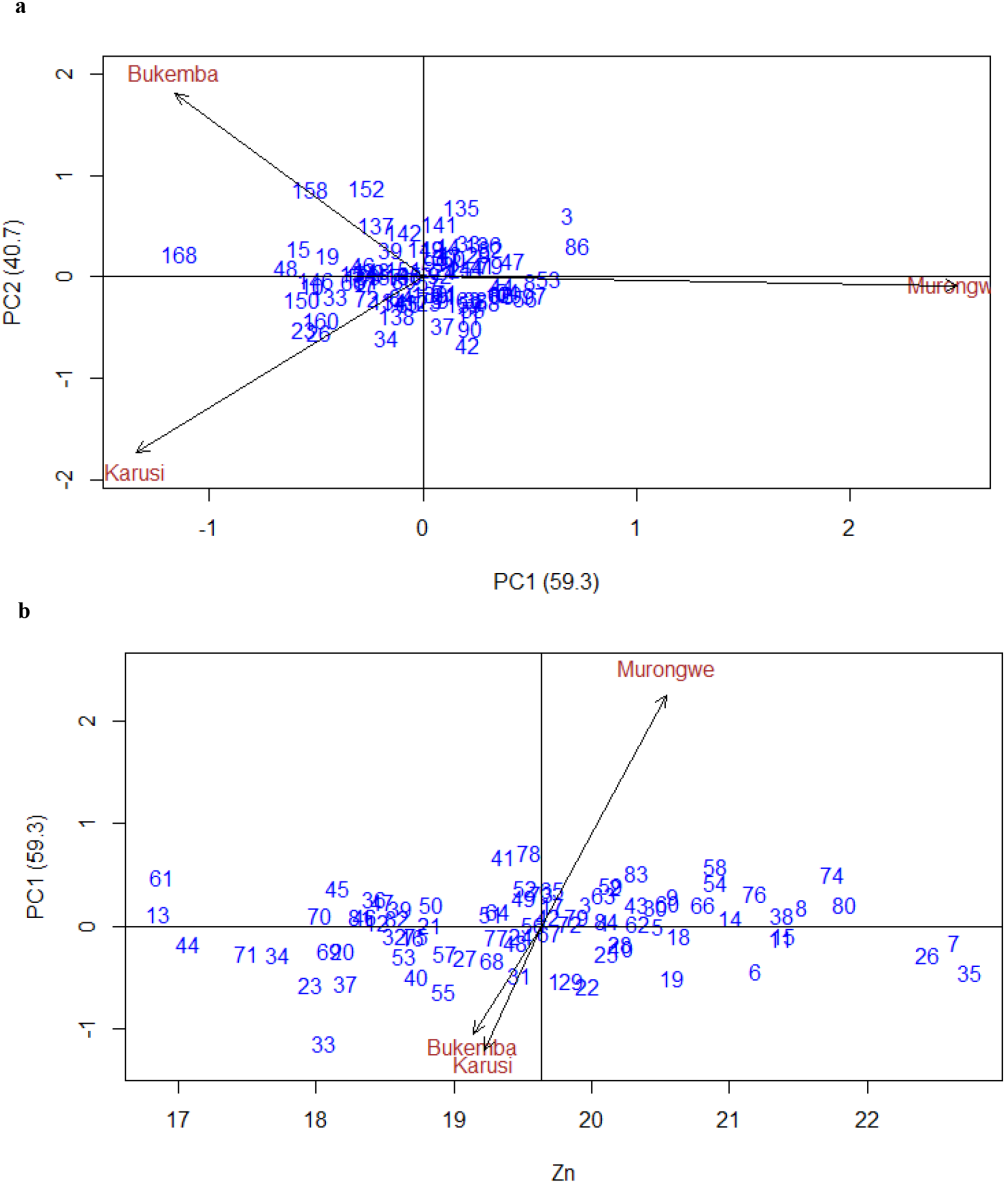
**(a)** GGE biplot for Zn PCA1 vs. PCA2 in climbing bean genotype across environments. **(b)** Bilpot for Zn of the climbing bean genotypes and environment IPCA score against means.

**Figure 8a** shows the performance and genotype-by-environment interactions (GEI) for Zn concentration in climbing bean genotypes across environments. Principal Component 1 (PC1) explained 59.3% of the total variation, reflecting the main genotype effects, while PC2 accounted for 40.7%, representing GEI together explaining 100% of the variability. **Figure 8b** also presents the interaction of climbing bean genotypes for Zn concentration across environments, using IPCA scores plotted against mean Zn content. PC1 explained 59.3% of the interaction variation. Genotypes located near the origin such as 50, 57, 77, 56, and 14 exhibited low interaction effects, indicating stable Zn performance and broad adaptability. In contrast, genotypes farther from the origin situated along the vectors, including 74, 80, 58, 83, 78, and 41 in Murongwe; 68, 55, 40, 33, 37, 23, 44, and 71 in Bukemba; and 31, 129, and 22 in Karusi, showed strong interaction effects, suggesting specific adaptation. Murongwe exhibited the highest interaction, suggesting it offers favorable conditions for maximizing Zn concentration in genotypes. Bukemba and Karusi showed lower interaction effects, indicating more consistent Zn performance across genotypes. Therefore, genotypes performing well in Murongwe may be prioritized for location-specific breeding, while stable genotypes are ideal candidates for multi-environment trials targeting broadly adapted biofortified varieties.

##### 3.1.7.3 GGE biplot for GYD_kgha^-1^ in climbing bean genotypes

**Figure 9:**
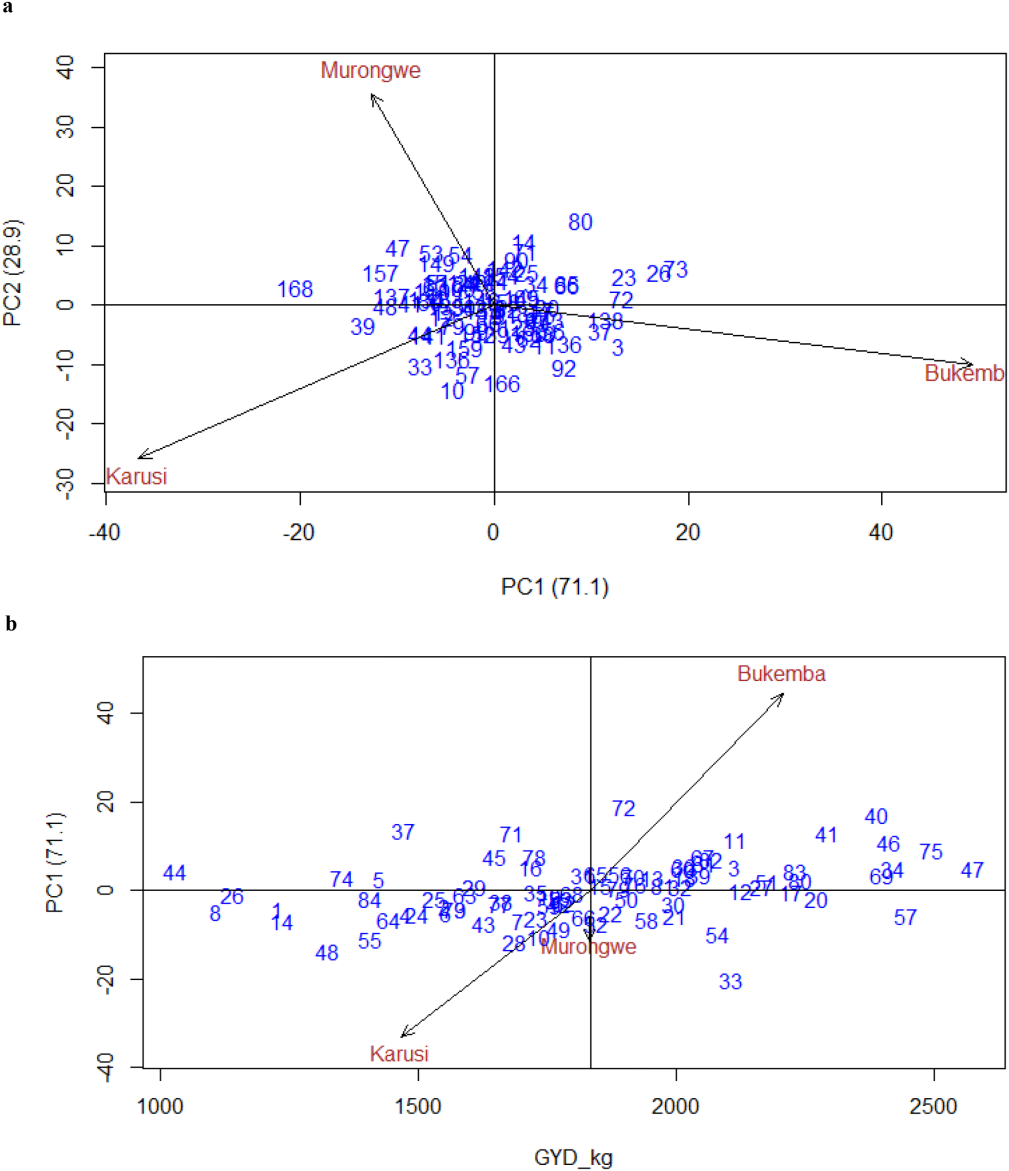
**(a)** GGE biplot for GYD_kgha^-1^ PCA1 vs. PCA2 climbing bean genotypes across environments. **(b)** Biplot for GYD_kgha-1 of climbing bean genotypes and environment IPCA score against means.

**Figures 9a, b** show GGE biplots for grain yield. In the **Figure 9a**, PC1 and PC2 explained 71.1% and 28.9% of the total variation, respectively, capturing 100% of the genotype-by-environment interaction. Murongwe and Karusi were positively correlated, while Bukemba showed contrasting responses. Moreover, **Figure 9b** illustrates the interaction of climbing bean genotypes for yield (kg ha^−1^) across environments, based on their IPCA scores plotted against mean yield, with PC1 accounting for 71.1% of the interaction variance. Genotypes located to the right of the biplot axis (e.g., 47, 75, 40, 46, 69, 24, 57, 41, 20, 34, 54) were high-yielding, while those on the left (e.g., 44, 8, 26, 1, 14, 48, 74, 5, 84, 6, 55) had lower yields. Genotypes clustered near the origin exhibited stable performance across environments. Conversely, genotypes positioned along specific environmental vectors such 72, 11, 41, 40, 46, 75, 47 in Bukemba; and 8, 14, 48, 55, 64, 43 in Karusi demonstrated strong GEI, indicating better performance in their respective environments.

## 4 Discussion

The highly significant environmental effects observed for all traits indicate that Fe, Zn, and grain yield expression were strongly influenced by site-specific conditions, consistent with variation in soil physicochemical properties and climatic factors across Murongwe, Bukemba, and Karusi (**Tables 1**–**3**). Such pronounced environmental influence on micronutrient accumulation and yield has similarly been reported in common bean and other grain legumes grown under diverse agroecological conditions (Tryphone and Nchimbi-Msolla, 2010).

The present study revealed substantial genetic variability for grain yield, iron (Fe), and zinc (Zn) concentrations among bush and climbing bean genotypes evaluated across contrasting environments in Burundi. The highly significant environmental effects (p < 0.001) observed for all traits confirm the strong influence of site-specific factors, including soil type, soil fertility status, temperature, rainfall distribution, and management practices, on micronutrient concentration and yield performance. These results are consistent with the documented soil characteristics and meteorological conditions of the experimental sites (**Tables 1**-**3**), which likely contributed to differential nutrient availability and crop responses. Similar environmental influences on Fe, Zn, and yield have been widely reported in common bean and other crops (Tryphone and Nchimbi-Msolla, 2010; Moraghan et al., 2002; Welch and Graham, 2002; Katuuramu et al., 2021).

Highly significant genotype effects for Fe, Zn, and grain yield in both bush and climbing beans highlighted the existence of considerable genetic variability for these traits. Similar variability in nutrient uptake and distribution in common beans was previously reported by Tryphone and Nchimbi-Msolla (2010). This genetic diversity forms the basis for effective selection and genetic improvement, aligning with breeding objectives aimed at enhancing productivity alongside nutritional quality. The presence of significant G×E interactions (p < 0.01) for most traits further demonstrates that genotype performance varied across environments, underlining the necessity of multi-environment trials to identify broadly adapted and stable genotypes. Such findings are in agreement with previous reports emphasizing the combined influence of genotype and environment on micronutrient accumulation and yield in common bean (Philipo et al., 2021, 2020).

The AMMI model revealed that the first two interaction principal components axis (PCA1 and PCA2) explained a large proportion of the G×E variation, with PCA1 accounting for the largest share, particularly for grain yield and micronutrient traits. This indicates that the main patterns of G×E interaction were captured by PCA1, while PCA2 explained residual interactions that were smaller and likely associated with minor environmental fluctuations or random effects. Similar observations were reported by Tryphone and Nchimbi-Msolla (2010), who documented large variability in Fe and Zn among genotypes and environments, emphasizing the complexity of biofortification efforts. The pronounced variability observed in Fe and Zn concentrations in this study suggests that successful genetic improvement must account for both genetic potential and environmental suitability, particularly under conditions of low soil fertility and climatic variability (**Tables 1**, **2**). These results are consistent with the study conducted by (Ejigu et al., 2024).

The first two principal components (PCA1 and PCA2) captured a substantial portion of the genotype × environment (G × E) interaction, with PCA1 contributing most to the explained variance, particularly for grain yield and micronutrient concentrations (**Table 4**). This indicates that PCA1 primarily reflected the systematic G × E effects, while PCA2 accounted for residual variation, which may be attributed to random or unexplained sources.

The mean performance and stability analysis using IPCA scores and AMMI’s Stability Value (ASV) further differentiated genotype responses within each growth habit. Among bush beans, Fe concentrations of the top 15 genotypes ranged from 52.86 to 76.5 ppm, values that are generally consistent with average Fe levels reported by Glahn et al. (2020). However, Zn concentrations were relatively low (17.42–23.10 ppm) compared with the wider range reported by Talukder et al. (2010). These comparatively low micronutrient levels are likely linked to environmental constraints, including acidic soils, suboptimal soil pH, uneven soil moisture, high temperatures during grain filling, and limited nutrient availability (**Tables 1**-**3**). Similar limiting effects of soil characteristics and crop nutrition on seed mineral accumulation have been reported by Welch and Graham (2002); Katuuramu et al. (2021); Mukamuhirwa and Rurangwa (2018), and Graham et al. (2001). Other studies have similarly shown that Fe and Zn concentrations are shaped by genetic factors and environmental conditions, including soil fertility, nutrient uptake efficiency, temperature, and related factors (Welch and Graham, 2002; Katuuramu et al., 2021). Comparable trends of low Fe and Zn content have also been reported in other crops such as pearl millet (Akhtar et al., 2023). In addition, Ligarreto–Moreno and Pimentel–Ladino (2022) indicated that common beans perform optimally at soil pH values between 5.5 and 6.5, whereas the experimental sites in this study were acidic, which may have constrained micronutrient availability. identifying genotypes with stable performance across environments aligns with recommendations for climate-resilient breeding (Manevska-Tasevska et al., 2023).

Despite these environmental limitations, several bush bean genotypes surpassed the check in Fe and Zn concentration, demonstrating their potential contribution to alleviating micronutrient deficiencies in Burundi. Genotype 27 recorded the highest yield but only moderate Fe and low Zn, emphasizing its suitability for yield-focused production systems rather than nutritional improvement. In contrast, genotype 167 combined relatively high yield with favorable Fe and Zn levels, making it particularly attractive for breeding programs targeting combined productivity and nutritional quality. Stability analysis revealed that some genotypes with low ASV values were stable but exhibited low yield or micronutrient content, reinforcing the assertion that stability alone is insufficient for selection. This observation supports findings by Philipo et al. (2021) and (Al-Naggar et al. (2018), who emphasized the need to consider both performance and stability in genotype selection.

In climbing beans, the top-performing genotypes exhibited Fe concentrations ranging from 53.38 to 59.7 ppm and Zn concentrations between 18.06 and 22.64 ppm, largely aligning with previously reported ranges (Glahn et al., 2020); Talukder et al., 2010). The relatively low micronutrient levels observed were again associated with environmental influences, particularly low soil fertility, low temperature, high humidity and pH conditions (**Tables 1**, **2**). These findings are consistent with reports by Bulyaba et al. (2020); Welch and Graham (2002); Katuuramu et al. (2021), who highlighted the importance of genotype–environment interactions in determining seed Fe and Zn content. Although some climbing genotypes showed high Fe and Zn concentrations, reduced yield was observed in certain cases, confirming the yield–micronutrient balance reported by Diaz et al. (2022). Stability analysis showed identified genotypes with lower ASV values as more stable and higher ASV values suggesting high sensitivity to environmental variation. These results are consistent with findings from other crops reported by Daemo and Ashango (2024); Maulana et al. (2020), and (Mohammadnia et al., 2021). Overall, the observed variation in Fe and Zn concentrations across bush and climbing beans and across environments supports findings by Mukamuhirwa and Rurangwa (2018), who attributed such differences to genetic diversity, nutrient uptake, and internal nutrient distribution. The fact that some bush bean genotypes exhibited higher mineral concentrations than climbing types suggests differences in mineral accumulation linked to growth habit and significant genotype-by-environment interactions, as also noted by Ligarreto (2023).

The multi-trait selection index (MTSI) complemented the AMMI analysis by integrating Fe, Zn, and grain yield into a single selection background. The AMMI approach (IPCA and ASV) effectively identified genotypes with high mean performance and stability across environments, but in several cases revealed stable genotypes with low yield or low micronutrient content. In contrast, the MTSI integrated grain yield, iron, and zinc simultaneously, prioritizing genotypes with balanced agronomic and nutritional performance. In bush beans, MTSI ranking was more strongly influenced by micronutrient density, whereas in climbing beans, yield contributed more heavily to index performance. These differences reflect inherent growth habit characteristics and support the need for habit-specific breeding strategies. The identification of genotypes such as 132 and 167 by both AMMI and MTSI confirmed their superiority in balancing yield, micronutrient content, and stability. Similar effectiveness of MTSI in identifying genotypes combining agronomic and quality traits has been reported by Ambrósio et al. (2024). Overall, combining MTSI with AMMI-based stability analysis offers a more robust framework for selecting bean genotypes that are productive, nutritionally enhanced, and suitable for diverse environments (Pour-Aboughadareh et al., 2025).

Broad-sense heritability estimates further clarified the genetic control of the studied traits. High heritability values for Fe and Zn in both bush and climbing beans indicated strong genetic control and favorable predictions for biofortification through selection. These estimates exceeded those reported by Diaz et al. (2022) and were consistent with findings by Lamptey et al. (2023); Phuke et al. (2017) and Mesera et al. (2022). Such high heritability suggested that Fe and Zn can be effectively improved through breeding. In contrast, grain yield exhibited low heritability and high environmental sensitivity, underscoring the importance of indirect selection and multi-environment testing for yield improvement. The greater phenotypic variance observed for Fe compared to Zn in both bush and climbing beans may be due to higher genetic diversity, stronger genotype × environment interactions, and greater sensitivity to environmental factors like soil pH and organic matter. These results align with Katuuramu et al. (2021), who emphasized the strong genetic influence on these traits, supporting their potential for selection and genetic improvement to enhance Fe nutrition.

Correlation analyses highlighted a consistently strong positive association between Fe and Zn in both growth habits, indicating shared genetic mechanisms governing mineral uptake and accumulation (Moraghan et al., 2002; Katuuramu et al., 2021). Therefore, improving one can enhance the other. Similar findings have been reported in beans and other crops like sorghum, rice, and chickpeas (Moraghan et al., 2002; Blair et al., 2009; Katuuramu et al., 2021; Ligarreto, 2023 and Ejigu et al., 2024), supporting their combined improvement in breeding programs.

Yield showed weak but significant negative correlations with both Fe and Zn, suggesting that increased yield slightly reduces Fe and Zn concentrations, possibly due to physiological or genetic constraints, consistent with Diaz et al. (2022), who highlighted this challenge in breeding. Conversely, Fe and Zn had a strong, highly significant positive correlation, indicating that improving one can enhance the other. Differences in trait correlations between bush and climbing beans suggested the need for distinct breeding strategies for each growth type. In bush beans, the weaker correlation between yield and micronutrient content facilitates simultaneous selection for high Fe, Zn, and yield. Comparable findings regarding the weak correlation between grain Fe and Zn contents and yield were reported by Phuke et al. (2017) and Ligarreto (2023). Elisabeth et al. (2021) noted that while efficient grain filling can boost yield, it may dilute Fe and Zn levels, explaining inverse relationships. In contrast, stronger yield and nutrient correlations in climbing beans call for a more balanced breeding approach to maintain nutritional quality. The consistently strong positive Fe–Zn correlation in both types supports simultaneous selection for both nutrients. (Katuuramu et al., 2021) also reported a positive correlation between Fe and Zn, attributing it to shared genetic mechanisms governing mineral transport and seed accumulation.

Finally, GGE biplot analyses effectively illustrated genotype performance, stability, and specific adaptation across environments. Environments differed in their discriminatory ability and representativeness emphasize the importance of careful site selection in breeding programs. Murongwe exhibiting strong interaction effects for micronutrients, while Bukemba often acted as a more stable and representative test environment. These findings are consistent with the interpretations of Haynes et al. (1998); Yan and Kang (2002) and Al-Naggar et al. (2018). The identification of both broadly adapted and specifically adapted genotypes provides valuable guidance for selection under diverse agro-ecological conditions.

## 5 Conclusion

This study s demonstrated that iron (Fe), zinc (Zn), and grain yield in both bush and climbing beans are significantly influenced by genotype, environment, and genotype-by-environment (G×E) interaction, confirming the combined role of genetic potential and site-specific conditions in trait expression. AMMI and GGE analyses indicated that the first two interaction principal components captured most G×E variation, effectively describing genotype responses across environments. Several bush and climbing genotypes exceeded check varieties for Fe and Zn, although higher micronutrient content was not consistently associated with superior yield. Stability analyses using IPCA scores and AMMI stability values distinguished broadly adapted genotypes from those with specific environmental responses, showing that stability alone does not guarantee high agronomic or nutritional performance. The multi-trait selection index (MTSI) successfully integrated yield, Fe, and Zn, identifying genotypes with balanced productivity and nutritional quality. Genotypes consistently favored by both stability and MTSI represent promising candidates for biofortification breeding. High broad-sense heritability for Fe and Zn, compared with lower heritability for yield, indicates stronger genetic control of micronutrient traits. Weak to moderate negative correlations between yield and micronutrients, alongside a strong positive Fe–Zn association, suggest that simultaneous improvement of Fe and Zn is feasible, although yield–nutrition balances must be considered. Overall, these findings underscore the value of multi-environment evaluation and integrated selection strategies for developing nutritionally enhanced common beans adapted to diverse agro-ecologies in Burundi.

(1) $$Y_{ge}=\mu +\alpha_{g}+\beta_{e}+\sum_{k=1}^{n}\varphi_{n}\gamma_{gn}\delta_{en}+\;\rho_{ge}$$

Where: $Y_{ge}$ represents the mean yield or mean iron or zinc content for the g^th^ genotype in the e^th^ environment; μ is the overall mean; $\alpha_{g}$ and $\;\beta_{e}$ are the genotype and environment deviations from the overall mean, respectively; $\gamma_{gn}$ and $\delta_{en}$ are the principal component scores for the genotype and environment along axis k; n is the maximum number of multiplicative terms; $\varphi_{n}$ is the k^th^ singular value of x (square root of the eigth value of xx’ or x’x); $\rho_{ge}$ is the error term.

(2) $$ASV\;=\;\sqrt{{\left(\frac{SS\;IPCA1}{SS\;IPCA2}X\;IPCA1\;Score\right)}^{2}}+(IPCA2\;Score)$$

Where ASV is the AMMI stability value, SS is the sum of squares, and IPCA1 and IPCA2 are the first and second interaction principal component axes, respectively. Larger IPCA scores (positive or negative) suggest that a genotype is more specifically adapted to certain environments. In contrast, smaller ASV scores indicate greater stability of a genotype across different environments.

(3) $$rX_{ij}=\;\frac{\theta_{ij}-\emptyset_{oj}}{\eta_{oj}\;-\;\phi_{oj}}\;x\;(\eta_{nj}-\phi_{nj})+\;\phi_{nj}$$

Where, $rX_{ij}$ is the rescaled value of the trait j for genotype i. It transforms the original trait values into a common scale (usually 0–100) for use in a multi-trait selection index. $\theta_{ij}$ and $\emptyset_{oj}$ are the new maximum and minimum values for trait j after rescaling, respectively. $\eta_{oj}$ and $\phi_{oj}$ represent the original maximum and minimum trait values, respectively.

(4) X= μ+Lf+ ϵ

Where Xis the vector of rescaled traits, μthe mean vector, Lthe factor loading matrix, fthe common factors, and ϵthe residuals.

(5) $$MTSI_{i}=\;\sqrt{\sum_{j=+1}^{f}{\left(\gamma_{ij}-\;\gamma_{j}\right)}^{2}}$$

Where MTSI_i_ is the multi-trait stability index for the ith genotype; ij γ is the score of the i^th^ genotype in the j^th^ factor (i = 1, 2,…, g; j = 1, 2,…, f); being g and f the number of genotypes and factors, respectively; $\gamma_{ij}$is the score of the i^th^ genotype and $\gamma_{j}$the ideotype score for the j^th^ factor.

(6) $$h^{2}=\frac{\sigma_{g}^{2}}{\left[\sigma_{g}^{2}+(\sigma_{g*l}^{2})+(\sigma_{e/rl}^{2})\right]}$$

Where $\sigma_{g}^{2}$= genotypic variance, representing variability among genotypes, ${\text{σ}}_{g*l}^{2}$ = genotype × location interaction variance, representing differential genotype responses across environments, l = number of environments (sites and/or seasons), r = number of replications within each environment and $\sigma_{e}^{2}$ is the error variance assuming all effects are random.

The variance components (**Equation 7**) were estimated using a mixed linear model, in which genotypes and their interactions were treated as random effects, while environments were considered either fixed or random.

(7) $$Y_{ijk}=\;A=\mu +\;E_{j\;}+GE_{ij}+R_{k\;}E_{J}+\;\epsilon_{ijk}$$

Where: $Y_{ijk}$= the observed trait value; μis the overall mean; $G_{i}$ = the random effect of the $i^{th}$genotype; $E_{j\;}$ = the effect of the $j^{th}$environment; $GE_{ij}$ = the genotype × environment interaction; $R_{k\;}E_{J}$ = the replication effect within environment; $\epsilon_{ijk}$ = the residual error


$$h^{2}=\;\frac{\sigma^{2}}{\sigma_{g}^{2}+\sigma_{ge}^{2}/nEnvs+\;\sigma_{e}^{2}/(nEnvs\;X\;nreps)\;+}nreps\sigma_{g}^{2}\sigma_{e}^{2}nEnvs\sigma_{ge}^{2}$$

## Data Availability

The original contributions presented in the study are included in the article/**Supplementary Material**. Further inquiries can be directed to the corresponding author.
