# Supplementary material for: Effect of the genetic and environment interaction on yield, Fe and Zn content among locally cultivated common bean (Phaseolus vulgaris L.) germplasm
Source: Front Plant Sci. 2026 Feb 20;17:1719270. doi: 10.3389/fpls.2026.1719270 (PMC12963333; doi:10.3389/fpls.2026.1719270)
Supplement: Supplementary file 1 [file DataSheet1.docx]

Supplementary Material

## Supplementary Tables

**APPENDIX A1: Details of experimental materials used for bush bean genotypes**

| **Genotype** | **Source** | **Market segment** |  | **Genotype** | **Source** | **Market segment** |
| --- | --- | --- | --- | --- | --- | --- |
| 1. Muyugubwe nain*^c^ | Burundi | Pink |  | 98. Amahunja*^b^ | Burundi | Yellow |
| 2. Urubonobono*^c^ | Burundi | Cream speckled |  | 101. Kibonobono*^a^ | Burundi | White speckled |
| 4. Amashigisha*^a^ | Burundi | Red |  | 102. Sinamino *^a^ | Burundi | Cream speckled |
| 6. Sinamakamwe*^c^ | Burundi | Beige |  | 103. Moree88002**^b^ | Burundi | Yellow |
| 7. Kajemunkangara*^b^ | Burundi | White speckled |  | 104. IZO201245**^a^ | IRAZ | Yellow |
| 13. Kiryabakwe*^a^ | Burundi | Yellow |  | 105. KATB1** | CIAT | Yellow |
| 16. Manyurane*^a^ | Burundi | Cream |  | 106. KATB9** | CIAT | Red |
| 18. Gisoda*^b^ | Burundi | Light brown |  | 107. KATX56**^a^ | CIAT | red |
| 21. Kameneke*^b^ | Burundi | Cream |  | 108. KATX69**^a^ | CIAT | Red mottled |
| 22. Magoma*^b^ | Burundi | Kablanket |  | 109. BISERA**^a^ | CIAT | Red mottled |
| 27. Sinamakamwe*^b^ | Burundi | Cream |  | 110. IZO2015110**^a^ | IRAZ | Yellow |
| 28. Urwera*^b^ | Burundi | White |  | 111. CODMLB003**^a^ | CIAT | Cream speckled |
| 29. Karabera*^b^ | Burundi | Cream |  | 112. RWR2154**^a^ | RAB | Cream speckled |
| 31. Kijumbura*a | Burundi | Red speckled |  | 113. RWR2245**^b^ | RAB | Red mottled |
| 32. Vondoro*^a^ | Burundi | Cream speckled |  | 114. BCB-11-315**^a^ | KARLO | Yellow |
| 35. Bufu**^a^ | Burundi | Yellow |  | 115. BCB-11-404**^b^ | KARLO | yellow |
| 40. Karasara*^b^ | Burundi | Beige |  | 116. RWR1092**^a^ | RAB | Red |
| 45. Dore de Kirundo**^a^ | Burundi | yellow |  | 117. ECDHR**^b^ | CIAT | beige |
| 49. Ubudida*^b^ | Burundi | Beige |  | 118. BFS18**^a^ | CIAT | red |
| 50. Marasira*^a^ | Burundi | Light brown |  | 119. BFS30**^b^ | CIAT | red |
| 52. Ruyange*^a^ | Burundi | Red |  | 120. BFS24**^b^ | CIAT | red |
| 59. Kalima*^a^ | Burundi | Red mottled |  | 121. BFS35***^b^ | CIAT | red |
| 61. Shushamazi*^b^ | Burundi | Red |  | 122. Musengo**^a^ | CIAT | Cream speckled |
| 64. Kwezikumwe*^b^ | Burundi | Beige |  | 123. Mukungugu**^b^ | CIAT | Light brown |
| 67. Mufyiri*^b^ | Burundi | Pink |  | 124. Musore**^b^ | INERA | Light brown |
| 68. NUS17***^a^ | CIAT | beige |  | 126. DAB939***^b^ | CIAT | Red mottled |
| 70. Amahatanyovu*^b^ | Burundi | Beige |  | 127. DAB580**^a^ | CIAT | Red mottled |
| 74. NUS13**^a^ | CIAT | Rose |  | 128. DAB581**^a^ | CIAT | Red mottled |
| 75. Shusha*^d^ | Burundi | Red |  | 129. DAB922***^a^ | CIAT | Cream speckled |
| 76. Agaharawe*^b^ | Burundi | Light brown |  | 130. Ibiserede*^a^ | Burundi | Red mottled |
| 77. Ncocere*^b^ | Burundi | White |  | 131. RCB593***^b^ | CIAT | Red |
| 78. Mabati*^b^ | Burundi | Cream |  | 132. KWC51***^b^ | CIAT | Cream speckled |
| 81. Ruryoha*^b^ | Burundi | Cream |  | 147. DAB570***^b^ | CIAT | Red mottled |
| 83. Kirinzara*^a^ | Burundi | Red |  | 155. Runyange1*^a^ | Burundi | Red mottled |
| 85. Amanyurane*^a^ | Burundi | Cream |  | 156. Sinamino 1*^a^ | Burundi | Cream speckled |
| 87. Rwandarugari*^b^ | Burundi | Red |  | 161. GLP2**^a^ | CIAT | Red mottled |
| 88. NUA225**^a^ | CIAT | Red mottled |  | 162. RWR2091**^a^ | RAB | Red |
| 89. Zimanumukwe*^b^ | Burundi | Brown |  | 163. KWP77***^a^ | CIAT | Red mottled |
| 93. Tsindinzara*^b^ | Burundi | Red |  | 164. IZO201560***^a^ | IRAZ | Yellow |
| 94. Pfahumvyagije*^b^ | Burundi | Beige |  | 165. Runyange2*^a^ | Burundi | Red mottled |
| 95. Kiryinkumi*^b^ | Burundi | Brown |  | 167. DAB576***^b^ | CIAT | Red mottled |
| 96. NUS31**^a^ | CIAT | Red |  |  |  |  |

**a = Kidney; b = Round; c = Oval; d = Plate; e = Trunqued; * = Landrace; ** = Released; *** = Pre-released**

**APPENDIX A2: Details of experimental materials used for climbing bean genotypes**

| **Genotype** | **Source** | **Market segment** |  | **Genotype** | **Source** | **Market segment** |
| --- | --- | --- | --- | --- | --- | --- |
| 3. Gahoro volubile*^a^ | Burundi | red |  | 69. Kameneke*^a^ | Burundi | Cream |
| 5. Umukutsa wera*^a^ | Burundi | White |  | 71. Yozofina*^b^ | Burundi | Beige speckled |
| 8. Ndimirinkobe*^b^ | Burundi | Light brown |  | 72. Sesekaza*^b^ | Burundi | white speckled |
| 9. Makutsa*^a^ | Burundi | Red |  | 73. Amasera*^a^ | Burundi | Cream speckled |
| 10. Runyenyeri*^b^ | Burundi | white |  | 79. Inakayoba*^b^ | Burundi | Yellow |
| 11. Ruvuzo*^a^ | Burundi | Kablankett |  | 80. Ruvyibushabatama*^a^ | Burundi | Cream |
| 12. Muyugubwe volubile*^a^ | Burundi | Cream |  | 82. Gipolisi*^d^ | Burundi | Light brown |
| 14. Buki*^b^ | Burundi | Cream |  | 84. Gaconge*^a^ | Burundi | Cream speckled |
| 15. Amavondoro*^b^ | Burundi | Cream speckled |  | 86. Mukutsa 2*^a^ | Burundi | red |
| 17. Kinure 1*^a^ | Burundi | Red |  | 90. Gasimbo*^a^ | Burundi | Red speckled |
| 19. Naruhengeri*^b^ | Burundi | Pink |  | 91. Vyuzura*^b^ | Burundi | Red |
| 20. Sinamino 2*^a^ | Burundi | Cream speckled |  | 92. Kiryabatama*^b^ | Burundi | Red |
| 23. Mamesa*a | Burundi | Yellow speckled |  | 97. Rusenyanzego**^b^ | Burundi | Yellow |
| 24. Nokia*^b^ | Burundi | Red |  | 99. Inyange*^b^ | Burundi | White |
| 25. Gihete*^b^ | Burundi | Red |  | 100. Pfahuntaye ntoya*^b^ | Burundi | Beige |
| 26. Kosorata*^a^ | Burundi | red Kablankett |  | 125. Pfahuntereye*^b^ | Burundi | Red |
| 30. Mvunakabago*^b^ | Burundi | Red |  | 133. NgwinxCab2***^a^ | CIAT | Cream speckled |
| 33. Rutondera*^a^ | Burundi | Red |  | 134. RWV1272**^b^ | RAB | Red |
| 34. Ibirambira*^a^ | Burundi | Red speckled |  | 135. AND10**^e^ | CIAT | White speckled |
| 36. Makutsapataro*^a^ | Burundi | Red |  | 136. Amatsinda 1*^b^ | Burundi | Light brown |
| 37. Kaje*^b^ | Burundi | Red |  | 137. GSZ611**^d^ | Burundi | cream |
| 38. Musosi*^a^ | Burundi | Beige |  | 138. MUHORO**^a^ | Burundi | Cream speckled |
| 39. Urwendengwe*^b^ | Burundi | White |  | 139. KINURE**^a^ | Burundi | Red cream |
| 41. Mugina*^a^ | Burundi | Yellow |  | 141. RWV1129**^a^ | CIAT | Kablanket |
| 42. Urutondera*^d^ | Burundi | White |  | 142. NAKAJE**^b^ | CIAT | Yellow speckled |
| 43. Maguru*^a^ | Burundi | Red |  | 143. Gisetsabagore**^a^ | Burundi | Yellow |
| 44. Magwabari*^a^ | Burundi | Yellow |  | 144. NOKIA**^b^ | Burundi | Yellow |
| 46. Burengeti*^a^ | Burundi | Kablanket |  | 145. NUV130**^b^ | CIAT | red |
| 47. Mwerasi*^a^ | Burundi | White |  | 146. NUV91**^b^ | CIAT | Red |
| 48. Rukoko*^e^ | Burundi | Cream |  | 148.IZO201543**^a^ | IRAZ | Red |
| 51. Kinure 2*^a^ | Burundi | Red cream |  | 149. G13607**^d^ | CIAT | Red |
| 53. Amatsinda 2*^a^ | Burundi | Light brown |  | 150. MAC70**^b^ | CIAT | Red mottled |
| 54. Kayoba*b | Burundi | Red cream |  | 151. GASILIDA**^b^ | CIAT | Pink |
| 55. Bwijurihe*^b^ | Burundi | Red |  | 152. VCB81013**^b^ | CIAT | white |
| 56. Reya*^b^ | Burundi | Red |  | 153. BIHOGO**^b^ | CIAT | Yellow speckled |
| 57. Nakabaya*^b^ | Burundi | Cream |  | 154. Vuninkingi**^b^ | CIAT | red |
| 58. Washonjutarandima*^d^ | Burundi | beige |  | 157. MAC13**^b^ | CIAT | Red mottled |
| 60. Amavunanzara*^b^ | Burundi | Cream |  | 158. MAC52**^b^ | CIAT | White speckled |
| 62. Mbagara*^b^ | Burundi | Yellow |  | 159. Mbagarumbise*^b^ | Burundi | Brown |
| 63. Namamesa*^b^ | Burundi | Yellow |  | 160. KWP75***^a^ | CIAT | Yellow |
| 65. Kaje ngufi*^b^ | Burundi | Cream speckled |  | 166. Mukwararaye*^b^ | Burundi | Red |
| 66. buryohe*^e^ | Burundi | beige |  | 168. MAC44**^b^ | CIAT | Red mottled |

**a = Kidney; b = Round; c = Oval; d = Plate; e = Trunqued; * = Landrace; ** = Released; *** = Pre-released**

**APPENDIX A3: Mean performance of the bush bean genotypes for yield (*GYD_kgha^-1^*), Fe and Zn content across environments, stability analysis using IPCA scores and AMMI's stability value (ASV)**

| Genotypes | **2024A** | | | **2024B** | | | **COMBINED SEASONS** | | | | | |
| --- | --- | --- | --- | --- | --- | --- | --- | --- | --- | --- | --- | --- |
|  | Fe | Zn | Yield | Fe | Zn | Yield | Fe | Zn | Yield | IPCA1 | IPCA2 | ASV |
| 1 | 41.5 | 17.21 | 1111.22 | 40.97 | 18.41 | 1738.89 | 41.24 | 17.81 | 1425.06 | -4.06 | 5.8 | 9.27 |
| 2 | 50.87 | 19.95 | 1113.56 | 47.55 | 20.08 | 2657.11 | 49.21 | 19.09 | 1885.33 | -6.3 | -1.64 | 11.33 |
| 4 | 50.06 | 18.42 | 993.33 | 48.42 | 19.22 | 2125.89 | 49.24 | 19.21 | 1559.61 | -3.5 | 0.19 | 6.23 |
| 6 | 53.21 | 17.05 | 1072.89 | 47.62 | 17.81 | 1997 | 50.41 | 19.23 | 1534.94 | 2.31 | 5.03 | 6.5 |
| 7 | 54.8 | 19.64 | 1336.44 | 51.94 | 18.9 | 1836.44 | 53.37 | 17.8 | 1586.44 | -5.22 | 1.19 | 9.37 |
| 13 | 43.34 | 16.93 | 838.33 | 43.35 | 18.07 | 1826.67 | 43.34 | 16.97 | 1332.5 | -11.56 | -6.46 | 21.57 |
| 16 | 46.04 | 18.88 | 1199.89 | 50.12 | 19.48 | 2320 | 48.08 | 17.31 | 1759.94 | -3.77 | 5.28 | 8.55 |
| 18 | 50.88 | 20.6 | 797.89 | 49.21 | 20.29 | 2232.67 | 50.05 | 17.19 | 1515.28 | -10.4 | -1.88 | 18.61 |
| 21 | 44.43 | 17.05 | 859.78 | 47.76 | 17.29 | 2461.56 | 46.1 | 18.72 | 1660.67 | 2.34 | -1.9 | 4.58 |
| 22 | 47.38 | 18.29 | 1375.22 | 47.71 | 18.18 | 2555.22 | 47.54 | 18.48 | 1965.22 | 5.22 | 10.26 | 13.84 |
| 27 | 63.67 | 16.47 | 1190.78 | 51.19 | 18.36 | 2269.22 | 57.43 | 14.71 | 1730 | 3.29 | -7.31 | 9.37 |
| 28 | 46.97 | 17.99 | 918.56 | 50.2 | 20.75 | 2161.78 | 48.58 | 15.41 | 1540.17 | -3.62 | -7.63 | 9.98 |
| 29 | 49.16 | 19.43 | 927.78 | 55.1 | 21.75 | 2228 | 52.13 | 18.36 | 1577.89 | -0.79 | 0.42 | 1.47 |
| 31 | 61.2 | 18.99 | 288.89 | 45.22 | 16.75 | 1432 | 53.21 | 19.13 | 860.44 | 2.19 | 0.88 | 4 |
| 32 | 56.12 | 20.53 | 756.11 | 49.61 | 19.25 | 1930 | 52.86 | 17.47 | 1343.06 | 4.08 | 3.37 | 8 |
| 35 | 49.9 | 18.44 | 735.44 | 53.94 | 20.74 | 1720.33 | 51.92 | 18.53 | 1227.89 | -12.44 | -13.52 | 25.94 |
| 40 | 51.55 | 16.68 | 1127.22 | 49.38 | 18.78 | 2322.33 | 50.46 | 18.63 | 1724.78 | 2.83 | 1.23 | 5.19 |
| 45 | 41.19 | 17.57 | 917.89 | 41.15 | 17.44 | 2265.56 | 41.17 | 18.64 | 1591.72 | -5.73 | -1.89 | 10.38 |
| 49 | 52.72 | 20.61 | 1136.56 | 48.53 | 21.35 | 1920.33 | 50.63 | 19.64 | 1528.44 | -3.76 | 4.21 | 7.9 |
| 50 | 50.42 | 19.14 | 1149.78 | 49.93 | 21.46 | 2182.67 | 50.17 | 19.29 | 1666.22 | -6.34 | 0.41 | 11.29 |
| 52 | 49.96 | 19.56 | 1376.67 | 43.14 | 19.43 | 2279.56 | 46.55 | 19.85 | 1828.11 | 1.86 | 4.56 | 5.64 |
| 59 | 49.79 | 18.49 | 1140 | 47.99 | 18.27 | 2178.56 | 48.89 | 18.73 | 1659.28 | 4.01 | 4.79 | 8.59 |
| 61 | 45.9 | 18.12 | 599.67 | 45.96 | 19.36 | 1712.56 | 45.93 | 20.97 | 1156.11 | -3.33 | -0.35 | 5.94 |
| 64 | 51.62 | 22.72 | 752.89 | 50.96 | 22.85 | 1889.22 | 51.29 | 19.02 | 1321.06 | -5.76 | 0.91 | 10.29 |
| 67 | 51 | 20.71 | 747 | 54.42 | 22.36 | 2116.33 | 52.71 | 19.53 | 1431.67 | -6.74 | 0.32 | 11.99 |
| 68 | 62.37 | 20.04 | 339.22 | 56.8 | 21.91 | 1450.78 | 59.58 | 17.86 | 895 | 3.4 | -1.24 | 6.18 |
| 70 | 46.04 | 15.78 | 2036 | 45.73 | 17.19 | 3400.67 | 45.88 | 18.79 | 2718.33 | 10.09 | 2.46 | 18.12 |
| 74 | 51.59 | 22.73 | 1349.67 | 55.76 | 22.11 | 1235.56 | 53.67 | 17.56 | 1292.61 | -0.02 | -3.95 | 3.95 |
| 75 | 56.83 | 18.97 | 590.78 | 45.12 | 19.53 | 2280.78 | 50.97 | 18.11 | 1435.78 | 1.35 | -5.69 | 6.18 |
| 76 | 55.91 | 21.79 | 786.67 | 51.39 | 19.69 | 2054.11 | 53.65 | 17.5 | 1420.39 | -2.82 | 1.19 | 5.16 |
| 77 | 62.89 | 19.72 | 514.78 | 49.49 | 19.66 | 1806.56 | 56.19 | 17.59 | 1160.67 | -0.32 | -5.18 | 5.21 |
| 78 | 52.22 | 17.3 | 1206.22 | 50.76 | 19.07 | 2347.78 | 51.49 | 20.23 | 1777 | 2.81 | -3.53 | 6.13 |
| 81 | 52.26 | 17.53 | 950.44 | 47.6 | 18.35 | 2141.44 | 49.93 | 22.87 | 1545.94 | -0.93 | 11.27 | 11.39 |
| 83 | 50.3 | 18.65 | 1192 | 46.8 | 19.01 | 2635.56 | 48.55 | 19.23 | 1913.78 | -6.64 | 2.34 | 12.04 |
| 85 | 57.19 | 18.85 | 1071 | 45.63 | 18.72 | 2284.44 | 51.41 | 17.58 | 1677.72 | 2.13 | 2.64 | 4.62 |
| 87 | 49.42 | 19.89 | 999.89 | 48.1 | 19.64 | 2212.33 | 48.76 | 19.57 | 1606.11 | 3.52 | 4.78 | 7.89 |
| 88 | 42.94 | 16.75 | 581.56 | 46.54 | 16.86 | 1927.78 | 44.74 | 19.18 | 1254.67 | -0.87 | -3.33 | 3.67 |
| 89 | 54.3 | 19.28 | 1317.44 | 50.42 | 20.22 | 1929.33 | 52.36 | 21.59 | 1623.39 | -3.67 | 9.32 | 11.38 |
| 93 | 51.98 | 18.68 | 1015.56 | 53.7 | 21.13 | 2565.11 | 52.84 | 17.38 | 1790.33 | 2.87 | 2.17 | 5.55 |
| 94 | 48.69 | 17.32 | 718.56 | 51.67 | 19.28 | 2330.44 | 50.18 | 18.25 | 1524.5 | 1.68 | -2.93 | 4.18 |
| 95 | 60.33 | 21.63 | 1101.44 | 46.68 | 20.04 | 2322.89 | 53.5 | 19.61 | 1712.17 | -3.39 | 8.29 | 10.25 |
| 96 | 54.91 | 21.15 | 209.67 | 54.03 | 19.49 | 1684.67 | 54.47 | 19.73 | 947.17 | -2.4 | -0.11 | 4.27 |
| 98 | 49.48 | 19.08 | 1109.67 | 49.27 | 19.28 | 1763.67 | 49.37 | 23.1 | 1436.67 | 5.03 | 3.31 | 9.55 |
| 101 | 46.09 | 19.25 | 985.11 | 46.89 | 18.94 | 2061.89 | 46.49 | 20.45 | 1523.5 | -0.6 | 4.17 | 4.3 |
| 102 | 51.77 | 17.79 | 1192.67 | 53.24 | 20.63 | 2021 | 52.51 | 20.01 | 1606.83 | -10.63 | 7.51 | 20.36 |
| 103 | 56.17 | 20.1 | 497.33 | 45.63 | 18.35 | 1916.56 | 50.9 | 17.17 | 1206.94 | -8.79 | 10.96 | 19.11 |
| 104 | 46.74 | 17.47 | 458.44 | 46.07 | 18.13 | 2132.67 | 46.41 | 18.23 | 1295.56 | -1.33 | -0.47 | 2.41 |
| 105 | 47.98 | 17.79 | 308.89 | 42.4 | 16.15 | 1374.89 | 45.19 | 17.42 | 841.89 | 10.98 | 0.11 | 19.55 |
| 106 | 54.36 | 18.43 | 182.67 | 37.93 | 16.19 | 1058.33 | 46.15 | 19.37 | 620.5 | -7.95 | 2.63 | 14.39 |
| 107 | 48.89 | 17.57 | 366.22 | 38.78 | 16.82 | 1162.56 | 43.83 | 20.59 | 764.39 | -9.38 | 0.09 | 16.7 |
| 108 | 48.32 | 19.28 | 503.78 | 46.07 | 18.16 | 1206.33 | 47.2 | 17.87 | 855.06 | 5.47 | 1.08 | 9.8 |
| 109 | 53.8 | 19.2 | 258.67 | 46.53 | 17.77 | 1795.22 | 50.16 | 19.89 | 1026.94 | 4.67 | 4.39 | 9.4 |
| 110 | 47.44 | 14.68 | 677 | 36.63 | 14.74 | 1801.33 | 42.03 | 19.59 | 1239.17 | 3.02 | -0.88 | 5.45 |
| 111 | 43.38 | 15.34 | 690.11 | 43.04 | 15.47 | 2343 | 43.21 | 18.82 | 1516.56 | 4.2 | 1.61 | 7.65 |
| 112 | 48.1 | 17.61 | 1097.11 | 45.42 | 19.1 | 2285.33 | 46.76 | 17.73 | 1691.22 | -4.5 | -0.01 | 8.01 |
| 113 | 56.25 | 18.71 | 995 | 50.12 | 19.54 | 2213.67 | 53.19 | 17.51 | 1604.33 | -3.45 | -1.1 | 6.24 |
| 114 | 54.84 | 17.48 | 224.33 | 44.45 | 17.46 | 2041.22 | 49.64 | 20.98 | 1132.78 | 3.47 | -0.33 | 6.19 |
| 115 | 53.94 | 18.55 | 426.56 | 48.69 | 18.51 | 1328.11 | 51.31 | 20.3 | 877.33 | 5.69 | -3.93 | 10.87 |
| 116 | 52.99 | 18.57 | 442.22 | 44.43 | 18.69 | 1749.22 | 48.71 | 19.49 | 1095.72 | -9.67 | -4.14 | 17.7 |
| 117 | 50.5 | 18.26 | 917.89 | 50.65 | 19.02 | 2269.11 | 50.57 | 18.38 | 1593.5 | 12 | -4.89 | 21.92 |
| 118 | 43.34 | 19.27 | 762.56 | 43.72 | 20.02 | 1864.44 | 43.53 | 17.43 | 1313.5 | 2.4 | 8.81 | 9.79 |
| 119 | 44.09 | 18.3 | 906.89 | 44.14 | 20.27 | 2367.78 | 44.11 | 18.74 | 1637.33 | 5.81 | -1.04 | 10.4 |
| 120 | 44.28 | 19.61 | 762.67 | 43.78 | 20.1 | 1723.78 | 44.03 | 22.79 | 1243.22 | 6.89 | -1.67 | 12.38 |
| 121 | 48.12 | 18.46 | 1171.78 | 44.89 | 18.99 | 2348.22 | 46.5 | 21.53 | 1760 | 15.49 | 0.05 | 27.57 |
| 122 | 49.51 | 20.27 | 866.78 | 49.73 | 21.67 | 1844.11 | 49.62 | 20.98 | 1355.44 | -0.15 | -11.55 | 11.55 |
| 123 | 45.98 | 17.96 | 1225.67 | 52.42 | 20.07 | 1943.33 | 49.2 | 19.27 | 1584.5 | -4.02 | -1.62 | 7.33 |
| 124 | 49.74 | 19.21 | 1179.78 | 50.48 | 19.84 | 1750.78 | 50.11 | 16.49 | 1465.28 | 5.34 | -2.07 | 9.73 |
| 126 | 46.89 | 18.19 | 656.44 | 43.36 | 17.53 | 2174.89 | 45.13 | 22.42 | 1415.67 | 6.7 | 0.34 | 11.93 |
| 127 | 56.16 | 19.32 | 678.78 | 47.03 | 18.26 | 2165.22 | 51.6 | 19.25 | 1422 | 0.4 | -9.93 | 9.95 |
| 128 | 51.88 | 17.43 | 539.56 | 48.73 | 17.7 | 2217.78 | 50.3 | 20.74 | 1378.67 | 3.76 | -0.61 | 6.72 |
| 129 | 49.98 | 18.69 | 540.78 | 46.89 | 17.54 | 1797 | 48.43 | 19.69 | 1168.89 | 1.06 | 3.44 | 3.92 |
| 130 | 48.99 | 17.97 | 282.44 | 43.17 | 17.21 | 1914.78 | 46.08 | 18.18 | 1098.61 | 1.99 | -2.58 | 4.39 |
| 131 | 44.37 | 20.24 | 1053.56 | 46.91 | 20.21 | 2305.22 | 45.64 | 17.94 | 1679.39 | 6.87 | 4.57 | 13.05 |
| 132 | 96.58 | 25.63 | 630.89 | 56.42 | 20.12 | 1602.89 | 76.5 | 18.83 | 1116.89 | -3.66 | -2.54 | 7 |
| 147 | 57.37 | 19.12 | 630.56 | 51.03 | 19.34 | 1859.22 | 54.2 | 18.79 | 1244.89 | -5.63 | -8.72 | 13.28 |
| 155 | 55.17 | 17.56 | 381.44 | 47.67 | 17.6 | 1733.78 | 51.42 | 19.77 | 1057.61 | -4.79 | 2.59 | 8.91 |
| 156 | 51.99 | 19.02 | 966.44 | 52.24 | 20.12 | 2351.89 | 52.12 | 16.81 | 1659.17 | -7.77 | 2.26 | 14.02 |
| 161 | 55.25 | 22.5 | 320.67 | 43.76 | 20.68 | 1468.33 | 49.51 | 19.75 | 894.5 | 1.91 | -4.64 | 5.75 |
| 162 | 49.38 | 18.14 | 419.56 | 43.8 | 16.62 | 2316.22 | 46.59 | 19.9 | 1367.89 | 5.8 | -9.87 | 14.29 |
| 163 | 49.31 | 18.93 | 295.78 | 43.5 | 17.57 | 1603.67 | 46.4 | 18.3 | 949.72 | 8.89 | -2.83 | 16.08 |
| 164 | 52.44 | 19.92 | 167.22 | 47.15 | 19.3 | 1774.78 | 49.8 | 20.84 | 971 | 5.34 | 1.13 | 9.58 |
| 165 | 56.05 | 19.91 | 557.11 | 53.83 | 19.54 | 1885.56 | 54.94 | 20.32 | 1221.33 | 4.58 | -9.32 | 12.39 |
| 167 | 67.32 | 22.64 | 1235.56 | 70.31 | 23.56 | 1802.56 | 68.82 | 19.18 | 1519.06 | 2.92 | 0.94 | 5.28 |

**APPENDIX A4: Mean performance of the climbing bean genotypes for yield (*GYD_kgha^-1^*), Fe and Zn content across environments, stability analysis using IPCA scores and AMMI's stability value (ASV)**

| **Genotypes** | **2024A** | | | **2024B** | | | **COMBINED SEASONS** | | | | | |
| --- | --- | --- | --- | --- | --- | --- | --- | --- | --- | --- | --- | --- |
|  | **Fe** | **Zn** | **Yield** | **Fe** | **Zn** | **Yield** | **Fe** | **Zn** | **Yield** | **IPCA1** | **IPCA2** | **ASV** |
| 3 | 41.83 | 18.92 | 1829.22 | 42.78 | 19.81 | 2757.78 | 42.31 | 19.36 | 2293.5 | 12.92 | -6.83 | 32.54 |
| 5 | 44.21 | 19.32 | 1419.22 | 46.69 | 19.82 | 2360.78 | 45.45 | 19.57 | 1890 | 3.67 | -1.55 | 9.17 |
| 8 | 53.45 | 20.85 | 840.56 | 54.19 | 22.64 | 1864.44 | 53.82 | 21.74 | 1352.5 | 2.99 | -1.07 | 7.44 |
| 9 | 47.04 | 19.82 | 1250.44 | 46.51 | 19.97 | 2524.78 | 46.78 | 19.89 | 1887.61 | 0.56 | -0.42 | 1.44 |
| 10 | 57.32 | 20.38 | 515.22 | 50.62 | 19.09 | 1943.33 | 53.97 | 19.74 | 1229.28 | -4.31 | -14.2 | 17.73 |
| 11 | 47.73 | 19.22 | 1720.33 | 45.56 | 20.7 | 2505.67 | 46.64 | 19.96 | 2113 | 5.18 | -6.59 | 14.35 |
| 12 | 55.55 | 19.89 | 752.11 | 50.17 | 20.29 | 2200.67 | 52.86 | 20.09 | 1476.39 | -5.42 | -1.97 | 13.5 |
| 14 | 42.11 | 16.48 | 1299 | 43.63 | 17.24 | 2608.22 | 42.87 | 16.86 | 1953.61 | 3.02 | 10.84 | 13.14 |
| 15 | 51.93 | 21.02 | 1408.67 | 52.82 | 18.92 | 2335.78 | 52.37 | 19.97 | 1872.22 | -5.18 | -1.05 | 12.79 |
| 17 | 42.94 | 17.64 | 1756.67 | 45.48 | 17.8 | 3079.56 | 44.21 | 17.72 | 2418.11 | 4.9 | -0.74 | 12.08 |
| 19 | 52.19 | 22.11 | 1177 | 53.77 | 23.38 | 2278.11 | 52.98 | 22.75 | 1727.56 | -0.35 | -1.75 | 1.96 |
| 20 | 39.26 | 18.09 | 1419.11 | 41.52 | 18.75 | 2613.22 | 40.39 | 18.42 | 2016.17 | 5.44 | -0.22 | 13.39 |
| 23 | 48.3 | 17.3 | 1393.67 | 49.55 | 19.12 | 1548.89 | 48.92 | 18.21 | 1471.28 | 13.48 | 4.98 | 33.56 |
| 24 | 54.2 | 20.93 | 836.78 | 48.47 | 21.84 | 2486 | 51.34 | 21.38 | 1661.39 | -2.57 | 4.05 | 7.52 |
| 25 | 49.99 | 19.32 | 1377.44 | 46.75 | 17.91 | 2710.33 | 48.37 | 18.61 | 2043.89 | 3.36 | 5.69 | 10.03 |
| 26 | 43.22 | 18.41 | 1831.78 | 43.51 | 19.06 | 2943.33 | 43.36 | 18.73 | 2387.56 | 17.04 | 5.52 | 42.32 |
| 30 | 45.99 | 19.4 | 1270.78 | 47.8 | 19.97 | 2271.22 | 46.9 | 19.69 | 1771 | -3.17 | -0.31 | 7.82 |
| 33 | 53.31 | 20.97 | 970.78 | 44.02 | 19.68 | 2283.44 | 48.67 | 20.33 | 1627.11 | -7.59 | -10.08 | 21.22 |
| 34 | 44.72 | 16.35 | 713.44 | 42.33 | 17.79 | 1345.33 | 43.53 | 17.07 | 1029.39 | 4.3 | 3.68 | 11.21 |
| 36 | 43.59 | 18.7 | 904.78 | 43.25 | 17.61 | 2390.33 | 43.42 | 18.16 | 1647.56 | 7.76 | -6.26 | 20.1 |
| 37 | 47.29 | 19.35 | 1829.56 | 43.41 | 17.34 | 2991.33 | 45.35 | 18.35 | 2410.44 | 11.01 | -4.19 | 27.42 |
| 38 | 46.39 | 18.38 | 2353.67 | 43.49 | 18.59 | 2797.33 | 44.94 | 18.49 | 2575.5 | 5.09 | -4.79 | 13.41 |
| 39 | 47.19 | 18.7 | 885.22 | 49.53 | 20.19 | 1764.11 | 48.36 | 19.45 | 1324.67 | -13.59 | -3.39 | 33.62 |
| 41 | 43.51 | 18.6 | 1067.89 | 49.36 | 20.42 | 2476.89 | 46.43 | 19.51 | 1772.39 | -8.62 | 0.48 | 21.22 |
| 42 | 45.64 | 18.17 | 963.11 | 47.96 | 19.51 | 2847.11 | 46.8 | 18.84 | 1905.11 | -2 | -0.29 | 4.94 |
| 43 | 44.7 | 18.39 | 1176 | 48.07 | 20.15 | 3177.22 | 46.39 | 19.27 | 2176.61 | 2.02 | -6.45 | 8.14 |
| 44 | 49.24 | 19.14 | 1505.56 | 44.58 | 19.9 | 2182.33 | 46.91 | 19.52 | 1843.94 | -7.55 | -4.68 | 19.16 |
| 46 | 42.19 | 17.91 | 1233.78 | 44.89 | 19.36 | 2319.33 | 43.54 | 18.64 | 1776.56 | -1.97 | 3.95 | 6.25 |
| 47 | 47.03 | 20.94 | 1760.67 | 45.36 | 20.85 | 2399.11 | 46.19 | 20.9 | 2079.89 | -9.89 | 9.78 | 26.25 |
| 48 | 45.76 | 18.77 | 838.78 | 48.94 | 19.09 | 1974.44 | 47.35 | 18.93 | 1406.61 | -11.2 | 0.07 | 27.57 |
| 51 | 44.67 | 19.19 | 1883.33 | 45.32 | 18.68 | 3007.44 | 45 | 18.94 | 2445.39 | -5.67 | 3.98 | 14.51 |
| 53 | 45.5 | 19.71 | 1621.89 | 48.12 | 22.09 | 2261.78 | 46.81 | 20.9 | 1941.83 | -6.49 | 9.03 | 18.35 |
| 54 | 54.47 | 21.01 | 1058.67 | 44.75 | 19.27 | 2447.44 | 49.61 | 20.14 | 1753.06 | -3.44 | 8.6 | 12.07 |
| 55 | 58.22 | 21.34 | 1042 | 45.34 | 19.77 | 2983.89 | 51.78 | 20.56 | 2012.94 | 5.1 | -1.31 | 12.62 |
| 56 | 48.78 | 16.3 | 1528.89 | 44.96 | 17.47 | 2579.56 | 46.87 | 16.89 | 2054.22 | 6.11 | -4.19 | 15.62 |
| 57 | 50.98 | 20.75 | 1040.44 | 46.7 | 19.92 | 2524.44 | 48.84 | 20.33 | 1782.44 | -2.76 | -11.44 | 13.31 |
| 58 | 49.49 | 21.42 | 1053.22 | 38.74 | 18.77 | 2128.22 | 44.11 | 20.09 | 1590.72 | -1.03 | 2.61 | 3.63 |
| 60 | 47.03 | 19.09 | 907.44 | 48.08 | 19.56 | 1979.67 | 47.55 | 19.32 | 1443.56 | -6.58 | 0.68 | 16.22 |
| 62 | 54.4 | 19.41 | 1591.33 | 53.57 | 20.02 | 2099.22 | 53.99 | 19.72 | 1845.28 | 3.67 | -5.35 | 10.51 |
| 63 | 47.55 | 21.84 | 1152.22 | 47.31 | 19.78 | 2488.89 | 47.43 | 20.81 | 1820.56 | -6.02 | 3.18 | 15.16 |
| 65 | 46.27 | 18.86 | 1756.67 | 48.2 | 20.53 | 2345.78 | 47.23 | 19.69 | 2051.22 | 7.53 | 3.84 | 18.93 |
| 66 | 46.77 | 19.04 | 1469.89 | 49.82 | 19.52 | 2125.11 | 48.3 | 19.28 | 1797.5 | -0.58 | -2.92 | 3.25 |
| 69 | 45.88 | 17.55 | 2068 | 46.58 | 18.65 | 2728.44 | 46.23 | 18.1 | 2398.22 | 3.37 | 1.46 | 8.41 |
| 71 | 48.67 | 17.43 | 1848.56 | 48.74 | 18.63 | 1985.89 | 48.71 | 18.03 | 1917.22 | 3.14 | 9.05 | 11.9 |
| 72 | 47.59 | 17.11 | 1215.22 | 44.51 | 17.87 | 2146.56 | 46.05 | 17.49 | 1680.89 | 13.08 | 1.14 | 32.21 |
| 73 | 49.92 | 19.13 | 1562.89 | 52.06 | 20.55 | 2233.67 | 50.99 | 19.84 | 1898.28 | 18.79 | 6.4 | 46.69 |
| 79 | 47.9 | 20.61 | 777 | 46.62 | 18.65 | 2365.89 | 47.26 | 19.63 | 1571.44 | -4.29 | -3.32 | 11.07 |
| 80 | 45.11 | 18.72 | 2245.89 | 42.8 | 18.74 | 2741.89 | 43.95 | 18.73 | 2493.89 | 9.01 | 14.23 | 26.36 |
| 82 | 46.63 | 20.19 | 1884 | 48.65 | 22.18 | 1954.44 | 47.64 | 21.19 | 1919.22 | 1.32 | -0.49 | 3.29 |
| 84 | 44.16 | 19.08 | 481.44 | 43.75 | 19.53 | 2837.44 | 43.95 | 19.31 | 1659.44 | -3.06 | 3.76 | 8.41 |
| 86 | 43.36 | 19.03 | 1199.67 | 44.62 | 20.07 | 2252.89 | 43.99 | 19.55 | 1726.28 | 7.51 | 3.43 | 18.81 |
| 90 | 57.12 | 21.93 | 1855.56 | 54.58 | 21.73 | 2628.22 | 55.85 | 21.83 | 2241.89 | 2.34 | 7.65 | 9.58 |
| 91 | 54 | 18.45 | 1075.22 | 52.41 | 18.21 | 2872.33 | 53.2 | 18.33 | 1973.78 | 1.23 | -1.46 | 3.36 |
| 92 | 54.6 | 19.13 | 1218.67 | 45.1 | 18.04 | 2914.89 | 49.85 | 18.59 | 2066.78 | 7.19 | -10.47 | 20.56 |
| 97 | 47.1 | 20.56 | 1391.56 | 48.12 | 20.11 | 3069.67 | 47.61 | 20.33 | 2230.61 | 4.46 | -2.4 | 11.23 |
| 99 | 50.79 | 19.69 | 898 | 52.37 | 20.52 | 1915.11 | 51.58 | 20.11 | 1406.56 | -1.87 | -4.13 | 6.19 |
| 100 | 46.43 | 19.6 | 1026.67 | 48.88 | 20.76 | 2075.22 | 47.66 | 20.18 | 1550.94 | -3.75 | 3.1 | 9.74 |
| 125 | 61.13 | 20.51 | 321 | 48.98 | 20.45 | 2531.33 | 55.06 | 20.48 | 1426.17 | 2.46 | -3.45 | 6.98 |
| 133 | 49.89 | 20.56 | 1183.44 | 52.17 | 21.83 | 1928.22 | 51.03 | 21.2 | 1555.83 | -5.01 | 1.07 | 12.37 |
| 134 | 55.96 | 22.79 | 911.44 | 53.35 | 22.49 | 2477.33 | 54.65 | 22.64 | 1694.39 | -6.85 | 1.05 | 16.89 |
| 135 | 54.33 | 23.57 | 710.67 | 49.04 | 19.49 | 1509.33 | 51.68 | 21.53 | 1110 | -4.85 | 0.02 | 11.94 |
| 136 | 48.71 | 20.64 | 1289.33 | 48.84 | 20.54 | 1878.11 | 48.77 | 20.59 | 1583.72 | -4.3 | -9.01 | 13.9 |
| 137 | 58.72 | 21.48 | 1096.22 | 48.03 | 18.94 | 2370.56 | 53.38 | 20.21 | 1733.39 | -10.48 | 1.61 | 25.85 |
| 138 | 48.16 | 21.3 | 2073.67 | 48.96 | 21.45 | 2153.33 | 48.56 | 21.37 | 2113.5 | 11.63 | -2.28 | 28.71 |
| 139 | 46.97 | 18.58 | 1428.78 | 43.24 | 18.31 | 2820.11 | 45.11 | 18.44 | 2124.44 | -0.23 | -4.6 | 4.63 |
| 141 | 59.17 | 20.79 | 844.89 | 59.35 | 21.23 | 1626.78 | 59.26 | 21.01 | 1235.83 | -6.78 | -5.03 | 17.44 |
| 142 | 58.32 | 20.56 | 1398.56 | 61.08 | 22.22 | 2305.56 | 59.7 | 21.39 | 1852.06 | 1.17 | 6.27 | 6.89 |
| 143 | 52.51 | 19.15 | 1341.11 | 46.68 | 18.25 | 2096.33 | 49.6 | 18.7 | 1718.72 | 5.4 | -2.46 | 13.52 |
| 144 | 48.7 | 19.87 | 1547.44 | 46.83 | 19.55 | 2896.22 | 47.77 | 19.71 | 2221.83 | -0.46 | 4.28 | 4.43 |
| 145 | 48.79 | 20.82 | 1495.89 | 48.98 | 20.44 | 2535.89 | 48.88 | 20.63 | 2015.89 | 2.94 | 1.39 | 7.38 |
| 146 | 53.07 | 21.34 | 725.89 | 50.41 | 19.83 | 2781.89 | 51.74 | 20.59 | 1753.89 | -1.26 | 0.97 | 3.25 |
| 148 | 38.88 | 17.43 | 1430.22 | 40.41 | 18.96 | 3112.56 | 39.65 | 18.19 | 2271.39 | -1.73 | 5.11 | 6.65 |
| 149 | 41.93 | 18.92 | 1038.11 | 39.35 | 18.74 | 2958.11 | 40.64 | 18.83 | 1998.11 | -5.81 | 7.23 | 16.03 |
| 150 | 51.95 | 17.49 | 910.11 | 52.73 | 18.43 | 2545.67 | 52.34 | 17.96 | 1727.89 | -6.32 | 2.54 | 15.77 |
| 151 | 51.25 | 19.15 | 1025.44 | 47.95 | 19.82 | 1972.56 | 49.6 | 19.49 | 1499 | -5.44 | 4.03 | 13.98 |
| 152 | 51.81 | 19.39 | 1029.67 | 56.73 | 20.82 | 2033 | 54.27 | 20.11 | 1531.33 | -1.76 | -4.74 | 6.42 |
| 153 | 56.05 | 22.78 | 587.56 | 52.32 | 22.1 | 1695.89 | 54.18 | 22.44 | 1141.72 | -1.09 | -0.64 | 2.76 |
| 154 | 43.75 | 18.58 | 1169.33 | 47.92 | 19.59 | 3162.67 | 45.84 | 19.09 | 2166 | 0.77 | 5.14 | 5.48 |
| 157 | 53.62 | 20.16 | 997 | 56.42 | 20.26 | 2379 | 55.02 | 20.21 | 1688 | -11.68 | 5.55 | 29.28 |
| 158 | 53.74 | 19.74 | 1168 | 57.25 | 19.97 | 2049.56 | 55.49 | 19.86 | 1608.78 | 0.96 | 0.78 | 2.48 |
| 159 | 56.71 | 21.04 | 1461.44 | 54.28 | 19.89 | 2530.44 | 55.5 | 20.47 | 1995.94 | -2.98 | -6.97 | 10.12 |
| 160 | 49.55 | 19.16 | 1390.89 | 44.06 | 19.79 | 2244.22 | 46.81 | 19.48 | 1817.56 | 3.61 | -4.58 | 9.99 |
| 166 | 43.22 | 18.77 | 1403.22 | 45.11 | 18.37 | 2609.78 | 44.16 | 18.57 | 2006.5 | 0.84 | -13.06 | 13.22 |
| 168 | 56.8 | 17.94 | 1478.89 | 52.97 | 18.17 | 2735.56 | 54.88 | 18.06 | 2107.22 | -20.37 | 2.94 | 50.22 |

**APPENDIX 5: Multi-trait selection for mean performance in bush bean genotypes**

| **Rank** | **Genotype** | **GH** | **GYD_kg** | **Fe** | **Zn** | **GYD_r** | **Fe_r** | **Zn_r** | **MTSI** |
| --- | --- | --- | --- | --- | --- | --- | --- | --- | --- |
| 1 | 167.DAB576 | Bush | 1519 | 68.8 | 23.1 | 0.428 | 0.783 | 1 | 0.612 |
| 2 | 132.KWC51 | Bush | 1117 | 76.5 | 22.9 | 0.237 | 1 | 0.973 | 0.764 |
| 3 | 95.Kiryinkumi | Bush | 1712 | 53.5 | 20.8 | 0.52 | 0.349 | 0.731 | 0.852 |
| 4 | 93.Tsindinzara | Bush | 1790 | 52.8 | 19.9 | 0.558 | 0.33 | 0.619 | 0.888 |
| 5 | 29.Karabera | Bush | 1578 | 52.1 | 20.6 | 0.456 | 0.31 | 0.701 | 0.928 |
| 6 | 67.Mufyiri | Bush | 1432 | 52.7 | 21.5 | 0.387 | 0.327 | 0.814 | 0.93 |
| 7 | 76.Agaharawe | Bush | 1420 | 53.7 | 20.7 | 0.381 | 0.353 | 0.719 | 0.938 |
| 8 | 74.Amasera | Bush | 1293 | 53.7 | 22.4 | 0.32 | 0.354 | 0.919 | 0.941 |
| 9 | 2.Urubonobono | Bush | 1885 | 49.2 | 20 | 0.603 | 0.228 | 0.632 | 0.943 |
| 10 | 89.Zimanumukwe | Bush | 1623 | 52.4 | 19.8 | 0.478 | 0.317 | 0.601 | 0.948 |
| 11 | 156.Sinamino_b2 | Bush | 1659 | 52.1 | 19.6 | 0.495 | 0.31 | 0.579 | 0.953 |
| 12 | 50.Marasira | Bush | 1666 | 50.2 | 20.3 | 0.498 | 0.255 | 0.666 | 0.958 |
| 13 | 49.Ubudida | Bush | 1528 | 50.6 | 21 | 0.433 | 0.268 | 0.747 | 0.96 |
| 14 | 7.Kajemunkangara | Bush | 1586 | 53.4 | 19.3 | 0.46 | 0.345 | 0.544 | 0.963 |
| 15 | 113.RWR2245 | Bush | 1604 | 53.2 | 19.1 | 0.469 | 0.34 | 0.527 | 0.97 |
| 16 | 64.Kwezikumwe | Bush | 1321 | 51.3 | 22.8 | 0.334 | 0.286 | 0.963 | 0.977 |
| 17 | 102.Sinamino_b1 | Bush | 1607 | 52.5 | 19.2 | 0.47 | 0.321 | 0.537 | 0.978 |
| 18 | 27.Sinamakamwe_b2 | Bush | 1730 | 57.4 | 17.4 | 0.529 | 0.46 | 0.323 | 0.986 |
| 19 | 18.Gisoda | Bush | 1515 | 50 | 20.4 | 0.427 | 0.251 | 0.684 | 0.995 |
| 20 | 85.Amanyurane | Bush | 1678 | 51.4 | 18.8 | 0.504 | 0.29 | 0.486 | 1.01 |
| 21 | 32.Vondoro | Bush | 1343 | 52.9 | 19.9 | 0.344 | 0.331 | 0.617 | 1.01 |
| 22 | 83.Kirinzara | Bush | 1914 | 48.6 | 18.8 | 0.616 | 0.209 | 0.491 | 1.02 |
| 23 | 165.Runyange_b2 | Bush | 1221 | 54.9 | 19.7 | 0.286 | 0.39 | 0.598 | 1.02 |
| 24 | 78.Mabati | Bush | 1777 | 51.5 | 18.2 | 0.551 | 0.292 | 0.414 | 1.02 |
| 25 | 77.Ncocere | Bush | 1161 | 56.2 | 19.7 | 0.257 | 0.425 | 0.594 | 1.02 |
| 26 | 68.NUS17 | Bush | 895 | 59.6 | 21 | 0.131 | 0.521 | 0.747 | 1.02 |
| 27 | 87.Rwandarugari | Bush | 1606 | 48.8 | 19.8 | 0.47 | 0.215 | 0.603 | 1.03 |
| 28 | 122.Musengo | Bush | 1355 | 49.6 | 21 | 0.35 | 0.239 | 0.746 | 1.03 |
| 29 | 16.Manyurane | Bush | 1760 | 48.1 | 19.2 | 0.543 | 0.196 | 0.534 | 1.04 |
| 30 | 52.Ruyange | Bush | 1828 | 46.6 | 19.5 | 0.576 | 0.152 | 0.57 | 1.04 |
| 31 | 124.Musore | Bush | 1465 | 50.1 | 19.5 | 0.403 | 0.253 | 0.574 | 1.05 |
| 32 | 147.DAB570 | Bush | 1245 | 54.2 | 19.2 | 0.298 | 0.369 | 0.539 | 1.05 |
| 33 | 75.Shusha | Bush | 1436 | 51 | 19.3 | 0.389 | 0.277 | 0.542 | 1.05 |
| 34 | 117.ECDHR | Bush | 1594 | 50.6 | 18.6 | 0.464 | 0.266 | 0.469 | 1.05 |
| 35 | 131.RCB593 | Bush | 1679 | 45.6 | 20.2 | 0.505 | 0.127 | 0.658 | 1.06 |
| 36 | 123.Mukungugu | Bush | 1584 | 49.2 | 19 | 0.46 | 0.227 | 0.513 | 1.06 |
| 37 | 22.Magoma | Bush | 1965 | 47.5 | 18.2 | 0.641 | 0.18 | 0.42 | 1.07 |
| 38 | 28.Urwera | Bush | 1540 | 48.6 | 19.4 | 0.438 | 0.21 | 0.556 | 1.07 |
| 39 | 127.DAB580 | Bush | 1422 | 51.6 | 18.8 | 0.382 | 0.295 | 0.487 | 1.07 |
| 40 | 4. Amashigisha | Bush | 1560 | 49.2 | 18.8 | 0.448 | 0.228 | 0.49 | 1.08 |
| 41 | 35.Bufu | Bush | 1228 | 51.9 | 19.6 | 0.29 | 0.304 | 0.582 | 1.08 |
| 42 | 40.Karasara | Bush | 1725 | 50.5 | 17.7 | 0.526 | 0.263 | 0.36 | 1.08 |
| 43 | 98.Amahunja | Bush | 1437 | 49.4 | 19.2 | 0.389 | 0.232 | 0.533 | 1.09 |
| 44 | 59.Kalima | Bush | 1659 | 48.9 | 18.4 | 0.495 | 0.218 | 0.438 | 1.09 |
| 45 | 121.BFS35 | Bush | 1760 | 46.5 | 18.7 | 0.543 | 0.151 | 0.479 | 1.1 |
| 46 | 94.Phahumvyagije | Bush | 1524 | 50.2 | 18.3 | 0.431 | 0.255 | 0.428 | 1.1 |
| 47 | 96.NUS31 | Bush | 947 | 54.5 | 20.3 | 0.156 | 0.376 | 0.669 | 1.1 |
| 48 | 81.Ruryoha | Bush | 1546 | 49.9 | 17.9 | 0.441 | 0.248 | 0.385 | 1.12 |
| 49 | 103.Moree82002 | Bush | 1207 | 50.9 | 19.2 | 0.28 | 0.275 | 0.539 | 1.12 |
| 50 | 112.RWR2154 | Bush | 1691 | 46.8 | 18.4 | 0.51 | 0.158 | 0.435 | 1.13 |
| 51 | 101.Kibonobono | Bush | 1524 | 46.5 | 19.1 | 0.43 | 0.151 | 0.523 | 1.13 |
| 52 | 119.BFS30 | Bush | 1637 | 44.1 | 19.3 | 0.485 | 0.0834 | 0.546 | 1.15 |
| 53 | 6.Sinamakamwe_b1 | Bush | 1535 | 50.4 | 17.4 | 0.436 | 0.262 | 0.325 | 1.15 |
| 54 | 161.GLP2 | Bush | 894 | 49.5 | 21.6 | 0.131 | 0.236 | 0.82 | 1.17 |
| 55 | 70.Amahatanyovu | Bush | 2718 | 45.9 | 16.5 | 1 | 0.133 | 0.212 | 1.17 |
| 56 | 128.DAB581 | Bush | 1379 | 50.3 | 17.6 | 0.361 | 0.259 | 0.34 | 1.18 |
| 57 | 164.IZO201560 | Bush | 971 | 49.8 | 19.6 | 0.167 | 0.244 | 0.584 | 1.2 |
| 58 | 118.BFS18 | Bush | 1314 | 43.5 | 19.6 | 0.33 | 0.0669 | 0.588 | 1.22 |
| 59 | 120.BFS24 | Bush | 1243 | 44 | 19.9 | 0.297 | 0.0809 | 0.613 | 1.22 |
| 60 | 21.Kameneke_b | Bush | 1661 | 46.1 | 17.2 | 0.496 | 0.139 | 0.294 | 1.22 |
| 61 | 116.RWR1092 | Bush | 1096 | 48.7 | 18.6 | 0.227 | 0.213 | 0.467 | 1.23 |
| 62 | 109.Bisera | Bush | 1027 | 50.2 | 18.5 | 0.194 | 0.255 | 0.45 | 1.23 |
| 63 | 129.DAB922 | Bush | 1169 | 48.4 | 18.1 | 0.261 | 0.206 | 0.406 | 1.24 |
| 64 | 155.Runyange_b1 | Bush | 1058 | 51.4 | 17.6 | 0.208 | 0.29 | 0.342 | 1.25 |
| 65 | 126.DAB939 | Bush | 1416 | 45.1 | 17.9 | 0.379 | 0.112 | 0.376 | 1.25 |
| 66 | 61.Shushamazi | Bush | 1156 | 45.9 | 18.7 | 0.255 | 0.135 | 0.48 | 1.25 |
| 67 | 115.BCB-11-404 | Bush | 877 | 51.3 | 18.5 | 0.122 | 0.287 | 0.456 | 1.25 |
| 68 | 104.IZO201245 | Bush | 1296 | 46.4 | 17.8 | 0.322 | 0.148 | 0.369 | 1.26 |
| 69 | 162.RWR2091 | Bush | 1368 | 46.6 | 17.4 | 0.356 | 0.153 | 0.318 | 1.26 |
| 70 | 114.BCB-11315 | Bush | 1133 | 49.6 | 17.5 | 0.244 | 0.24 | 0.329 | 1.26 |
| 71 | 31.Kijumbura | Bush | 860 | 53.2 | 17.9 | 0.114 | 0.341 | 0.377 | 1.27 |
| 72 | 45.Dore de Kirundo | Bush | 1592 | 41.2 | 17.5 | 0.463 | 0 | 0.333 | 1.32 |
| 73 | 108.KATX69 | Bush | 855 | 47.2 | 18.7 | 0.112 | 0.171 | 0.478 | 1.32 |
| 74 | 13.Kiryabakwe | Bush | 1332 | 43.3 | 17.5 | 0.339 | 0.0616 | 0.333 | 1.33 |
| 75 | 130.Ibiserede | Bush | 1099 | 46.1 | 17.6 | 0.228 | 0.139 | 0.343 | 1.33 |
| 76 | 163.KWP77 | Bush | 950 | 46.4 | 18.3 | 0.157 | 0.148 | 0.422 | 1.33 |
| 77 | 1.Muyugubwe_b | Bush | 1425 | 41.2 | 17.8 | 0.384 | 0.00195 | 0.37 | 1.33 |
| 78 | 88.NUA225 | Bush | 1255 | 44.7 | 16.8 | 0.302 | 0.101 | 0.25 | 1.36 |
| 79 | 111.CODMLB003 | Bush | 1517 | 43.2 | 15.4 | 0.427 | 0.0578 | 0.0836 | 1.43 |
| 80 | 105.KATB1 | Bush | 842 | 45.2 | 17 | 0.106 | 0.114 | 0.269 | 1.46 |
| 81 | 106.KATB9 | Bush | 620 | 46.1 | 17.3 | 0 | 0.141 | 0.31 | 1.49 |
| 82 | 107.KATX56 | Bush | 764 | 43.8 | 17.2 | 0.0686 | 0.0754 | 0.296 | 1.49 |
| 83 | 110.IZO2015110 | Bush | 1239 | 42 | 14.7 | 0.295 | 0.0245 | 0 | 1.56 |

**APPENDIX 6: Multi-trait selection for mean performance in Climbing bean genotypes**

| **Rank** | **Genotype** | **GH** | **GYD_kg** | **Fe** | **Zn** | **GYD_r** | **Fe_r** | **Zn_r** | **MTSI** |
| --- | --- | --- | --- | --- | --- | --- | --- | --- | --- |
| 1 | 90.Gasimbo | Climbing | 2242 | 55.8 | 21.8 | 0.784 | 0.808 | 0.845 | 0.328 |
| 2 | 142.Nakaje | Climbing | 1852 | 59.7 | 21.4 | 0.532 | 1 | 0.77 | 0.521 |
| 3 | 159.Mbagarumbise | Climbing | 1996 | 55.5 | 20.5 | 0.625 | 0.79 | 0.613 | 0.578 |
| 4 | 134.RWV1272 | Climbing | 1694 | 54.7 | 22.6 | 0.43 | 0.748 | 0.982 | 0.623 |
| 5 | 19.Naruhengeri | Climbing | 1728 | 53 | 22.7 | 0.452 | 0.665 | 1 | 0.643 |
| 6 | 55.Bwijurihe | Climbing | 2013 | 51.8 | 20.6 | 0.636 | 0.605 | 0.628 | 0.653 |
| 7 | 138. Muhoro | Climbing | 2114 | 48.6 | 21.4 | 0.701 | 0.444 | 0.767 | 0.672 |
| 8 | 145.NUV130 | Climbing | 2016 | 48.9 | 20.6 | 0.638 | 0.46 | 0.641 | 0.742 |
| 9 | 15.Amavondoro | Climbing | 1872 | 52.4 | 20 | 0.545 | 0.634 | 0.529 | 0.75 |
| 10 | 62.Mbagara | Climbing | 1845 | 54 | 19.7 | 0.528 | 0.715 | 0.486 | 0.754 |
| 11 | 157.MAC13 | Climbing | 1688 | 55 | 20.2 | 0.426 | 0.767 | 0.57 | 0.754 |
| 12 | 146.NUV91 | Climbing | 1754 | 51.7 | 20.6 | 0.469 | 0.603 | 0.634 | 0.758 |
| 13 | 24.Nokia_c1 | Climbing | 1661 | 51.3 | 21.4 | 0.409 | 0.583 | 0.769 | 0.76 |
| 14 | 137.GSZ611 | Climbing | 1733 | 53.4 | 20.2 | 0.455 | 0.685 | 0.57 | 0.762 |
| 15 | 97.Rusenyanzego | Climbing | 2231 | 47.6 | 20.3 | 0.777 | 0.397 | 0.59 | 0.762 |
| 16 | 82. Gipolisi | Climbing | 1919 | 47.6 | 21.2 | 0.576 | 0.398 | 0.735 | 0.782 |
| 17 | 73.Amasera | Climbing | 1898 | 51 | 19.8 | 0.562 | 0.566 | 0.507 | 0.79 |
| 18 | 47.Mwerasi | Climbing | 2080 | 46.2 | 20.9 | 0.679 | 0.326 | 0.686 | 0.809 |
| 19 | 144.NOKIA_c2 | Climbing | 2222 | 47.8 | 19.7 | 0.771 | 0.405 | 0.485 | 0.82 |
| 20 | 158.MAC52 | Climbing | 1609 | 55.5 | 19.9 | 0.375 | 0.79 | 0.509 | 0.822 |
| 21 | 53.Amatsinda | Climbing | 1942 | 46.8 | 20.9 | 0.59 | 0.357 | 0.686 | 0.825 |
| 22 | 133.NgwinxCab2 | Climbing | 1556 | 51 | 21.2 | 0.34 | 0.568 | 0.737 | 0.831 |
| 23 | 63.Namamesa | Climbing | 1821 | 47.4 | 20.8 | 0.512 | 0.388 | 0.672 | 0.849 |
| 24 | 57.Nakabaya | Climbing | 1782 | 48.8 | 20.3 | 0.487 | 0.458 | 0.591 | 0.851 |
| 25 | 152.VCB81013 | Climbing | 1531 | 54.3 | 20.1 | 0.325 | 0.729 | 0.552 | 0.855 |
| 26 | 54.Kayoba | Climbing | 1753 | 49.6 | 20.1 | 0.468 | 0.497 | 0.558 | 0.855 |
| 27 | 11.Ruvuzo | Climbing | 2113 | 46.6 | 20 | 0.701 | 0.349 | 0.527 | 0.859 |
| 28 | 8.Ndimirinkobe | Climbing | 1352 | 53.8 | 21.7 | 0.209 | 0.707 | 0.83 | 0.861 |
| 29 | 125.Pfahuntereye | Climbing | 1426 | 55.1 | 20.5 | 0.257 | 0.768 | 0.616 | 0.868 |
| 30 | 65.Kaje Ngufi | Climbing | 2051 | 47.2 | 19.7 | 0.661 | 0.378 | 0.482 | 0.878 |
| 31 | 168.MAC44 | Climbing | 2107 | 54.9 | 18.1 | 0.697 | 0.76 | 0.204 | 0.885 |
| 32 | 91.Vyuzura | Climbing | 1974 | 53.2 | 18.3 | 0.611 | 0.676 | 0.25 | 0.905 |
| 33 | 12.Muyugubwe_c | Climbing | 1476 | 52.9 | 20.1 | 0.289 | 0.659 | 0.549 | 0.909 |
| 34 | 141.RWV1129 | Climbing | 1236 | 59.3 | 21 | 0.134 | 0.978 | 0.705 | 0.916 |
| 35 | 136.Amatsinda_1 | Climbing | 1584 | 48.8 | 20.6 | 0.359 | 0.455 | 0.634 | 0.918 |
| 36 | 33.Rutondera | Climbing | 1627 | 48.7 | 20.3 | 0.387 | 0.45 | 0.59 | 0.921 |
| 37 | 9.Makutse | Climbing | 1888 | 46.8 | 19.9 | 0.555 | 0.355 | 0.516 | 0.921 |
| 38 | 92.Kiryabatama | Climbing | 2067 | 49.8 | 18.6 | 0.671 | 0.509 | 0.294 | 0.921 |
| 39 | 43.Maguru | Climbing | 2177 | 46.4 | 19.3 | 0.742 | 0.336 | 0.41 | 0.925 |
| 40 | 66.Buryohe | Climbing | 1798 | 48.3 | 19.3 | 0.497 | 0.431 | 0.411 | 0.961 |
| 41 | 25.Gihete | Climbing | 2044 | 48.4 | 18.6 | 0.656 | 0.435 | 0.299 | 0.964 |
| 42 | 44.Magwabari | Climbing | 1844 | 46.9 | 19.5 | 0.527 | 0.362 | 0.452 | 0.965 |
| 43 | 154.Vuninkingi | Climbing | 2166 | 45.8 | 19.1 | 0.735 | 0.309 | 0.379 | 0.966 |
| 44 | 99.Inyange | Climbing | 1407 | 51.6 | 20.1 | 0.244 | 0.595 | 0.552 | 0.968 |
| 45 | 153.Bihogo | Climbing | 1142 | 54.2 | 22.4 | 0.0727 | 0.725 | 0.948 | 0.969 |
| 46 | 30.Mvunakabago | Climbing | 1771 | 46.9 | 19.7 | 0.48 | 0.361 | 0.481 | 0.974 |
| 47 | 160.KWP75 | Climbing | 1818 | 46.8 | 19.5 | 0.51 | 0.357 | 0.445 | 0.981 |
| 48 | 51.Kinure_c2 | Climbing | 2445 | 45 | 18.9 | 0.916 | 0.267 | 0.353 | 0.981 |
| 49 | 100.Pfahuntaye Ntoya | Climbing | 1551 | 47.7 | 20.2 | 0.337 | 0.399 | 0.565 | 0.995 |
| 50 | 5.Umukutsa wera | Climbing | 1890 | 45.5 | 19.6 | 0.557 | 0.289 | 0.461 | 0.996 |
| 51 | 41.Mugina | Climbing | 1772 | 46.4 | 19.5 | 0.481 | 0.338 | 0.451 | 1 |
| 52 | 143.Gisetsabagore | Climbing | 1719 | 49.6 | 18.7 | 0.446 | 0.496 | 0.313 | 1.02 |
| 53 | 42.Urutondera | Climbing | 1905 | 46.8 | 18.8 | 0.566 | 0.357 | 0.337 | 1.02 |
| 54 | 151.Gasilida | Climbing | 1499 | 49.6 | 19.5 | 0.304 | 0.496 | 0.447 | 1.02 |
| 55 | 38.Musosi | Climbing | 2576 | 44.9 | 18.5 | 1 | 0.264 | 0.277 | 1.03 |
| 56 | 37.Kaje | Climbing | 2410 | 45.4 | 18.3 | 0.893 | 0.284 | 0.254 | 1.04 |
| 57 | 80.Ruvyibushabatama | Climbing | 2494 | 44 | 18.7 | 0.947 | 0.215 | 0.318 | 1.04 |
| 58 | 79.Inakayoba | Climbing | 1571 | 47.3 | 19.6 | 0.351 | 0.379 | 0.471 | 1.04 |
| 59 | 69.Kameneke_c | Climbing | 2398 | 46.2 | 18.1 | 0.885 | 0.328 | 0.211 | 1.04 |
| 60 | 150.MAC70 | Climbing | 1728 | 52.3 | 18 | 0.452 | 0.633 | 0.188 | 1.05 |
| 61 | 10.Runyenyeri | Climbing | 1229 | 54 | 19.7 | 0.129 | 0.714 | 0.489 | 1.05 |
| 62 | 135.AND10 | Climbing | 1110 | 51.7 | 21.5 | 0.0521 | 0.6 | 0.794 | 1.05 |
| 63 | 3.Gahoro volubile | Climbing | 2294 | 42.3 | 19.4 | 0.818 | 0.133 | 0.426 | 1.06 |
| 64 | 71.Yozofina | Climbing | 1917 | 48.7 | 18 | 0.574 | 0.452 | 0.2 | 1.06 |
| 65 | 26.Kosorata | Climbing | 2388 | 43.4 | 18.7 | 0.878 | 0.185 | 0.319 | 1.07 |
| 66 | 139.Kinure-c3 | Climbing | 2124 | 45.1 | 18.4 | 0.708 | 0.272 | 0.27 | 1.07 |
| 67 | 86.Mukutsa2 | Climbing | 1726 | 44 | 19.6 | 0.451 | 0.216 | 0.458 | 1.1 |
| 68 | 58.Washonje utarandima | Climbing | 1591 | 44.1 | 20.1 | 0.363 | 0.223 | 0.55 | 1.1 |
| 69 | 166.Mukwararaye | Climbing | 2006 | 44.2 | 18.6 | 0.632 | 0.225 | 0.291 | 1.11 |
| 70 | 60.Amavunanzara | Climbing | 1444 | 47.6 | 19.3 | 0.268 | 0.394 | 0.419 | 1.11 |
| 71 | 39.Urwedengwe | Climbing | 1325 | 48.4 | 19.4 | 0.191 | 0.434 | 0.44 | 1.14 |
| 72 | 84.Gaconge | Climbing | 1659 | 44 | 19.3 | 0.408 | 0.215 | 0.416 | 1.14 |
| 73 | 17.Kinure_c1 | Climbing | 2418 | 44.2 | 17.7 | 0.898 | 0.227 | 0.147 | 1.16 |
| 74 | 48.Rukoko | Climbing | 1407 | 47.3 | 18.9 | 0.244 | 0.384 | 0.352 | 1.17 |
| 75 | 23.Mamesa | Climbing | 1471 | 48.9 | 18.2 | 0.286 | 0.463 | 0.231 | 1.18 |
| 76 | 46.Burengeti | Climbing | 1777 | 43.5 | 18.6 | 0.483 | 0.194 | 0.302 | 1.18 |
| 77 | 149.G13607 | Climbing | 1998 | 40.6 | 18.8 | 0.627 | 0.0495 | 0.335 | 1.22 |
| 78 | 56.Reya | Climbing | 2054 | 46.9 | 16.9 | 0.663 | 0.36 | 0.00556 | 1.23 |
| 79 | 72.Sesekaza | Climbing | 1681 | 46 | 17.5 | 0.421 | 0.319 | 0.108 | 1.26 |
| 80 | 20.Sinamino | Climbing | 2016 | 40.4 | 18.4 | 0.638 | 0.037 | 0.266 | 1.26 |
| 81 | 36.Makutsapataro | Climbing | 1648 | 43.4 | 18.2 | 0.4 | 0.188 | 0.221 | 1.28 |
| 82 | 148.IZO201543 | Climbing | 2271 | 39.6 | 18.2 | 0.803 | 0 | 0.227 | 1.28 |
| 83 | 14.Buki | Climbing | 1954 | 42.9 | 16.9 | 0.598 | 0.161 | 0 | 1.37 |
| 84 | 34.Ibirambira | Climbing | 1029 | 43.5 | 17.1 | 0 | 0.193 | 0.0359 | 1.61 |
